# The Influence of Synaptic Weight Distribution on Neuronal Population Dynamics

**DOI:** 10.1371/journal.pcbi.1003248

**Published:** 2013-10-24

**Authors:** Ramakrishnan Iyer, Vilas Menon, Michael Buice, Christof Koch, Stefan Mihalas

**Affiliations:** Allen Institute for Brain Science, Seattle, Washington, United States of America; Indiana University, United States of America

## Abstract

The manner in which different distributions of synaptic weights onto cortical neurons shape their spiking activity remains open. To characterize a homogeneous neuronal population, we use the master equation for generalized leaky integrate-and-fire neurons with shot-noise synapses. We develop fast semi-analytic numerical methods to solve this equation for either current or conductance synapses, with and without synaptic depression. We show that its solutions match simulations of equivalent neuronal networks better than those of the Fokker-Planck equation and we compute bounds on the network response to non-instantaneous synapses. We apply these methods to study different synaptic weight distributions in feed-forward networks. We characterize the synaptic amplitude distributions using a set of measures, called tail weight numbers, designed to quantify the preponderance of very strong synapses. Even if synaptic amplitude distributions are equated for both the total current and average synaptic weight, distributions with sparse but strong synapses produce higher responses for small inputs, leading to a larger operating range. Furthermore, despite their small number, such synapses enable the network to respond faster and with more stability in the face of external fluctuations.

## Author Summary

Neurons communicate via action potentials. Typically, depolarizations caused by presynaptic firing are small, such that many synaptic inputs are necessary to exceed the firing threshold. This is the assumption made by standard mathematical approaches such as the Fokker-Planck formalism. However, in some cases the synaptic weight can be large. On occasion, a single input is capable of exceeding threshold. Although this phenomenon can be studied with computational simulations, these can be impractical for large scale brain simulations or suffer from the problem of insufficient knowledge of the relevant parameters. Improving upon the standard Fokker-Planck approach, we develop a hybrid approach combining semi-analytical with computational methods into an efficient technique for analyzing the effect that rare and large synaptic weights can have on neural network activity. Our method has both neurobiological as well as methodological implications. Sparse but powerful synapses provide networks with response celerity, enhanced bandwidth and stability, even when the networks are matched for average input. We introduce a measure characterizing this response. Furthermore, our method can characterize the sub-threshold membrane potential distribution and spiking statistics of very large networks of distinct but homogeneous populations of 10s to 100s of distinct neuronal cell types throughout the brain.

## Introduction

Experiments analyzing the distribution of synaptic weights impinging onto neurons typically observe low-amplitude peaks with only few, large-amplitude excitatory (EPSPs) or inhibitory post-synaptic potentials (IPSPs) [1]–[14]. These have been fitted by lognormal [1], truncated Gaussian [10], [11] or highly skewed non-Gaussian distributions [4], [6], [9], [13]. This raises the question of the functional role of such relatively rare but powerful synaptic inputs. The functional implications of such strong synapses can be very significant [15]. *Circuits of the Mind* [16] proposes a powerful computational brain architecture (the neuroidal model) to explain the brain's remarkable flexibility to quickly memorize new events and associate them with previous stored ones. It is based on a very small fraction of powerful excitatory synapses. Furthermore, a recent study [17] of strong but sparse synapses, combined with weak and probabilistic synaptic amplitude distributions provided both computational justification as well as empirical support for the role of these rare yet powerful synaptic events in supporting low-frequency, spontaneous firing in neuronal networks at rest. This question has become more acute following several reports that individual cortical pyramidal neurons from human tissue recovered during surgery are sufficiently powerful to drive other neurons by themselves [18], [19], unlike equivalent cells in rodent cortex.

The relation between probabilistic synaptic weight distribution and population dynamics can be studied using simulations [17]. However, the large parameter space to be explored and the need to repeat simulations many times makes this an impractical first method to apply, in particular when modeling the activity in large regions or even the entire mammalian brain. An alternative is the analytical Fokker-Planck method, based on a continuous stochastic process (a brief review of stochastic processes following [20] and [21]–[23] has been provided in Text (S1)) that models the dynamics of homogeneous neuronal populations with a single partial differential equation (PDE). It is able to quickly explore parameter space and provide analytical insights [24] and characterizes any distribution of synaptic weights by just 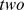 quantities-the drift and diffusion terms in the equation, corresponding to what the mean and variance of the membrane potential would be if the neuron did not have a threshold. But this method cannot reproduce dynamics in the presence of large synapses. Models based on jump stochastic processes [25], [26] treat synaptic input as being composed of pulses with finite amplitudes. The population dynamics in the presence of such synapses have been investigated using analytical and numerical techniques [27]–[38]. Richardson and Swarbrick [39] characterized analytically an integral formulation in the case of exponentially distributed stochastic jump processes. We here present a fast, semi-analytical approach to study the integral formulation of stochastic jump processes with arbitrary distributions. We apply this method to study the role of synaptic distributions on neuronal population dynamics.

## Results

### Mathematical formulation

The equation for the membrane potential 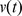 relative to rest for a generalized leaky integrate and fire (gLIF) neuron with normalized capacitance is,
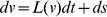
(1)

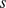
 is a random variable characterizing the shot-noise synaptic current. It takes the value 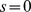 with probability 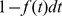 and 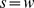 with probability 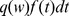. 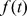 is the input synaptic event rate so that 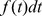 is the mean number of inputs in a time 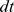. 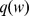 represents the distribution of synaptic weights, with 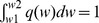 and 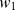 and 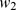 being the minimum and maximum synaptic weights respectively. When the synaptic input is sufficient to cause the membrane potential to exceed a threshold value 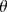, it is reset so that
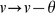
(2)This reset implementation is used to account for the shot-noise nature of the synaptic input. For nearly instantaneous synapses, 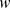 represents the peak of the post-synaptic potential (either EPSPs or IPSPs) (for non-instantaneous synapses, see Methods: Non-instantaneous synapses). 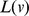 represents the sum of all non-synaptic currents, which can be voltage-dependent but not explicitly time-dependent. For the standard LIF neuron, 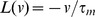, where 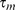 is the membrane time constant. For an exponential integrate-and-fire neuron, 
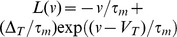
. 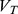 is the spike detection threshold and 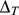 is the slope factor. The resulting equation for the probability 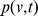 of a neuron to have a voltage 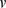 in 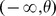 at time 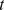-the master equation-is an integro-partial differential equation with displacement (DiPDE),
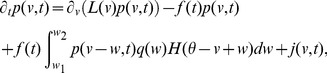
(3)with
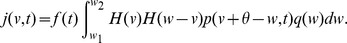


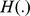
 is the unit step function and 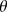 the threshold membrane potential.

The first term on the RHS in equation (3) incorporates the drift due to non-synaptic currents. The second term removes the probability for neurons which previously were at potential 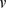 and received a synaptic input. The third term adds the probability that a neuron 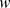 away in potential receives a synaptic input such that its potential is changed to 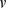. The last term 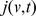 represents a *probability* current injection of the neuron which previously spiked. 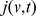 includes the effect of any excess synaptic input above the threshold at which spiking occurs, due to large super-threshold synaptic events. To account for the effect of the excess input with instantaneous synapses, the probability current is injected between the resting potential and 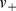, where 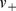 represents the membrane potential that would be reached due to the excess input. This serves the purpose of ‘remembering’ the excess input, whose effect would have been held by the synaptic variables in the case of slow synapses, without losing it on resetting the membrane potential after spiking. The output firing rate is given by
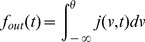
(4)In the Fokker-Planck formalism, 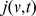 is part of a boundary condition. If 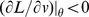, then there is no probability current through threshold due to continuous processes for this system. The alternative case is discussed in (Methods: Boundary conditions). Since we include non-infinitesimal synaptic inputs in an infinitesimal time interval, it is possible for a neuron to cross the threshold by a large amount. In equation (3) , this additional depolarization is accounted for in the term 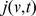 as outlined above. The case in which this additional depolarization is ignored is treated in (Methods: Boundary conditions). Simpifications obtained with 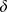-function distribution of synaptic weights 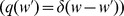 are described in (Methods: Simplifications). It is also possible to include the effect of synaptic delays and distributions of synaptic delays within the DiPDE formalism, as discussed in (Methods: Non-instantaneous synapses).

The stationary solution for equation (3) can be obtained as the solution to the following equation,
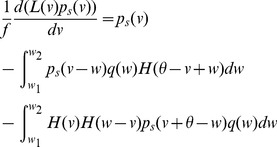
(5)where 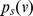 is the stationary probability distribution for the membrane potential.

Although the model can include both excitatory and inhibitory connections, all the results presented below, except for those in Figure S6 c) and d), are for feed-forward networks with excitatory connections.

### Comparison to Fokker-Planck formalism and simulations

Intuitively, this model quickly diverges from the Fokker-Planck formalism when the synaptic strength is large. Consider the hypothetical case of a neuron starting at rest which has all the synapses equal and large. After a short time step, the Fokker-Planck formalism produces a narrow Gaussian distribution in membrane potential near rest, while the DiPDE formalism yields a membrane potential distribution which is a sum of two scaled delta functions: a large one at rest, and a small one at the synaptic weight. Over time, the Fokker-Planck equation converges to a single broader Gaussian, while the DiPDE formalism leads to a larger coefficient for the delta function at the synaptic weight value. A generalization to conductance-based synapses is presented in (Methods: Conductance-based synapses), while a generalization to the case of exponential integrate-and-fire neurons [40] is presented in (Methods: Exponential integrate-and-fire neurons).

**Figure 1:**
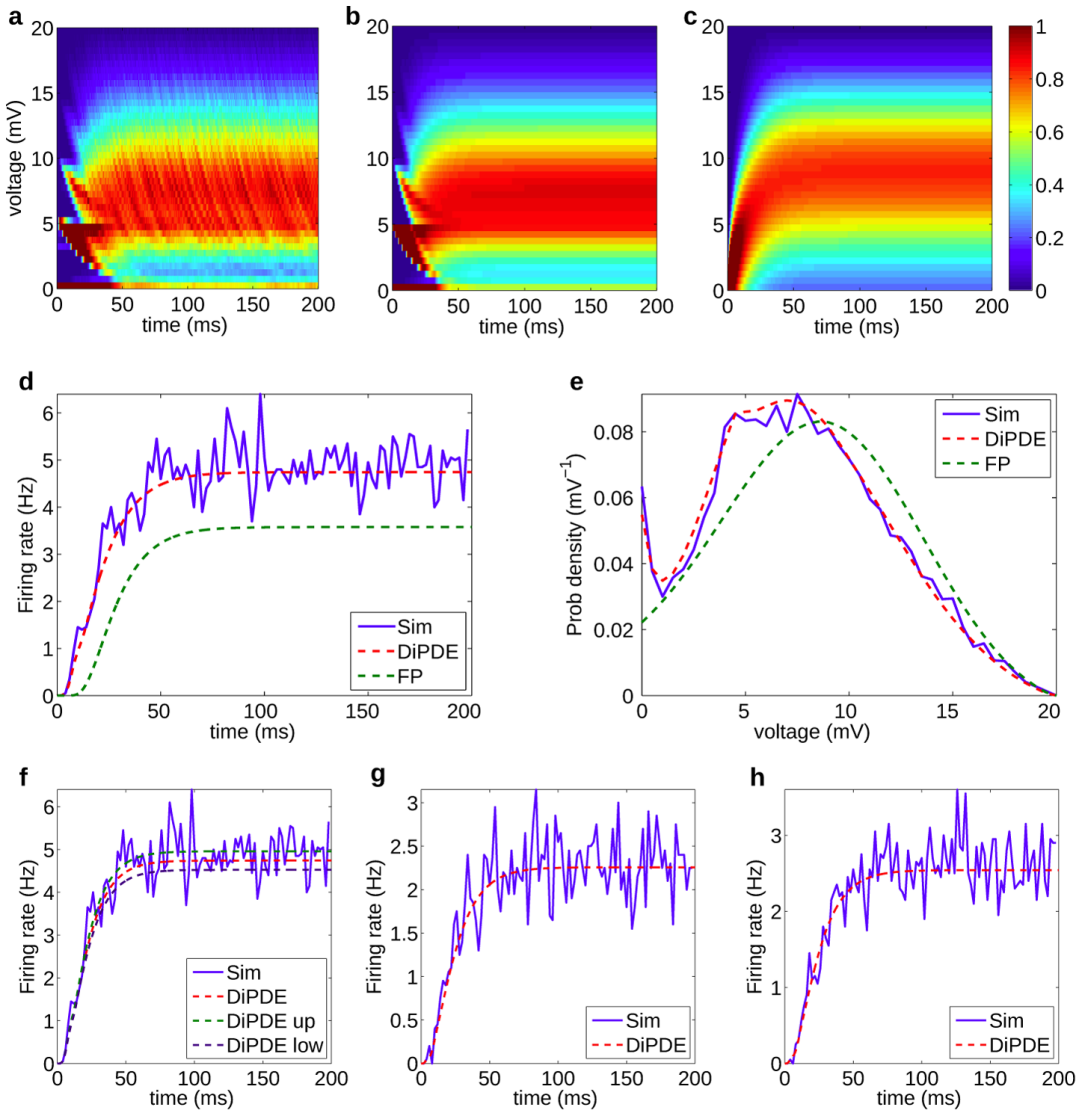
Comparisons between simulations and DiPDE. Panels (a)–(e) show results for excitatory, low-frequency, large amplitude current-based synapses of constant weight. Topmost panels show time evolution of the probability distribution of membrane potentials in the neuronal population obtained with Poisson input for 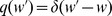 with synaptic weight (maximum EPSP) 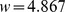 mV and input rate 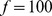 Hz from, a) simulations of 10,000 leaky integrate-and-fire (LIF) neurons (see Methods: Population Simulations for parameters used in simulations) , b) the numerical solution to the DiPDE equation (3), and c) the Fokker-Planck (FP) equation. Middle panels: d) Output firing rates as a function of time. e) The distribution of the sub-threshold steady-state membrane potential after 200 ms. These discrete synaptic jumps are evident in the voltage distributions just after synaptic input is switched on. Bottom panels: f) Expected 95% intervals for spike counts obtained from DiPDE for simulation data shown in panels (a)–(e). g) Output firing rates obtained from equation (21) for leaky integrate-and-fire (LIF) neurons and equivalent numerical simulations (see Methods: Population Simulations for parameters used in simulations), for excitatory conductance-based synapses. Poisson input for 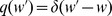 with maximum depolarization achieved by a neuron starting from rest 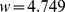 mV and input rate 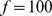 Hz. h) Output firing rates obtained from equation (21) for exponential integrate-and-fire (EIF) neurons and equivalent numerical simulations (see Methods: Population Simulations for parameters used in simulations), for excitatory conductance-based synapses without adaptation. Poisson input for 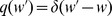 with maximum depolarization achieved by a neuron starting from rest 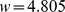 mV and input rate 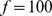 Hz.

We solve equation (3) without the displacement terms using the method of characteristics. The characteristic equations can be solved to obtain a non-uniform discretization of the membrane potential. For the standard LIF neuron, the characteristic equations are solved analytically. We then numerically add the effects of the displacement terms at every time step (see Methods: Numerical Solutions). This semi-analytic technique has the advantage of reducing errors due to numerical diffusion at each time-step. The solution of the DiPDE equation (3) is in good agreement with simulations of 10,000 leaky integrate-and-fire neurons for both low frequency, large amplitude (Figure (1)) and for high frequency, small amplitude distributions (Figure (S1)). Differences between continuous and discontinuous stochastic processes can be seen in the transient behavior of the probability distribution of membrane potential in the top panel (Figure 1a,b,c) and are statistically significant. p-values for differences between the sub-threshold steady-state membrane potential distributions have been provided in Text (S4), Table (S2) and Table (S3).

The steady state firing rate obtained from DiPDE is 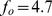 Hz and from simulations is 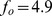 Hz. The small discrepancy is caused in part by simulating synapses which are not instantaneous but rather have a time constant of 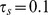 ms (for more details see (Methods: Non-instantaneous synapses)). Since the stochastic input is Poisson-distributed, expected 95% intervals for spike counts can be directly computed from DiPDE (see Methods: Expected 95% intervals for spike counts and Figure (1f)). The transient time to firing, defined as the time taken to reach 10% of the equilibrium firing rate 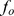, is 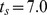 ms and provides a good estimate of how quickly the neuronal population responds to a given input.

By comparison, the Fokker-Planck formalism results in a lower steady state firing rate of 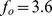 Hz and a slower transient time 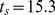 ms. The higher number of neurons closer to resting potential at equilibrium in Figure (1e) reflects the nature of the ‘jump’ stochastic process. For higher-frequency, low-amplitude inputs, all three methods converge to the same results (Text (S2) and Figure (S1)). Thus, the formalism presented in equation (3) is in good agreement with simulations for instantaneous synaptic input and does not depend on the choice of time-step for the numerical solution (Figure (S2)). For non-instantaneous synapses, upper and lower bounds on the steady state output firing rates can be obtained (Methods: Non-instantaneous synapses, Figure (S4) and Figure (S5)). The DiPDE implementation also matches equivalent simulations for conductance-based synapses (Figure (1g), Figure (S3)) and exponential integrate and fire neurons (Figure (1h)).

### Effect of synaptic weight distribution on population dynamics

**Figure 2:**
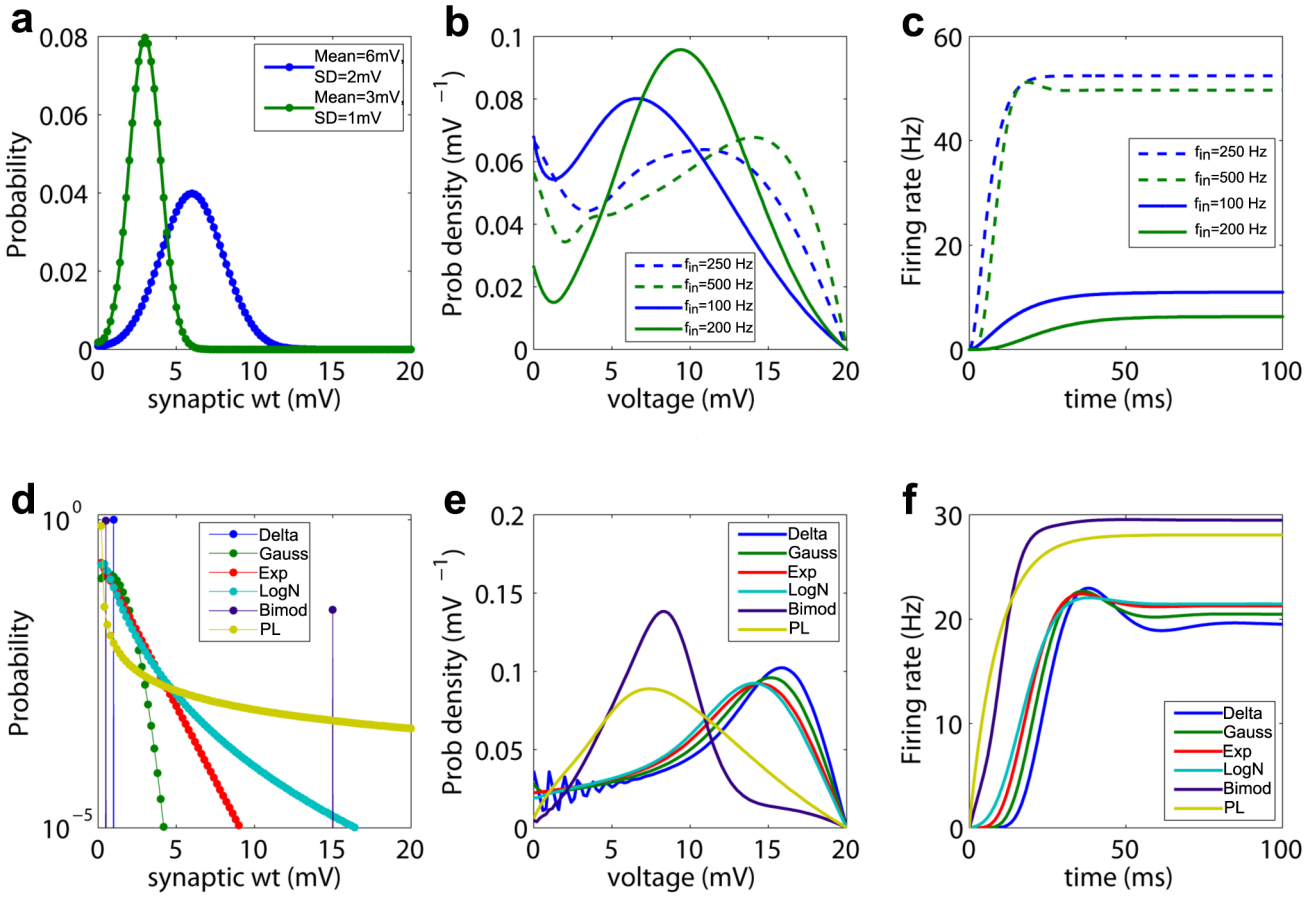
Distributions of instantaneous excitatory synaptic weights with same mean input current. Top panels: a) Two self-similar Gaussian synaptic weight distributions. b) The distribution of the sub-threshold, steady-state membrane potential when the two Gaussian synaptic inputs are activated with either a low (solid curves) or a high input firing rate (dashed curves) adjusted such that the mean input currents are equal. The low-amplitude distribution always has twice the input rate of the high amplitude one. In the absence of a threshold, these synaptic input would depolarize 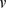 by 12 mV and 30 mV respectively. As we use a threshold of 20 mV, these inputs lead to distinct results, with the first being driven primarily by variations in input and the second by the mean input. c) Output firing rates as a function of time. In b) and c), green curves correspond to the Gaussian distribution with mean (3 mV) and standard deviation (SD) of (1 mV) and blue curves correspond to the Gaussian distribution with mean (6 mV) and SD (2 mV). For equal currents, stronger synapses produce a quicker response and a higher equilibrium firing rate. Bottom panels: d) Semi-log plot of 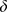-function (Delta), Gaussian (Gauss), exponential (Exp), lognormal (LogN), bi-modal (BiMod) and power-law (PL) synaptic weight distributions, matched for mean weight (1 mV) (see Methods: Matched distributions for the exact forms used for these distributions). e) Steady-state, sub-threshold voltage distributions and f) output firing rates for an input rate of 1,000 Hz. Heavier-tailed distributions produce quicker transients.

We used the DiPDE formalism to investigate the effect of generic synaptic weight distribution on the steady-state, subthreshold membrane potential distribution and output firing rates in feed-forward networks. Gaussian synaptic weight distributions with different mean weights whose input firing rates are adjusted to produce the same average synaptic current, result in different transient and steady-state firing rates as well as different equilibrium voltage distributions (top row in Figure (2), balanced excitation/inhibition Figure (S6)).

We then examined six different distributions tuned to have both the same mean (1 mV) synaptic weight and input rate (1,000 Hz), so that the average synaptic current was the same (see Methods: Matched synaptic distributions for the exact forms of the distributions and their respective variances). We find that the average synaptic current is not sufficient to accurately determine either the output firing rate or membrane voltage distributions (bottom row in Figure (2)). We also tested distributions that were matched to have input synaptic current with the same mean and variance (Figure (S7)). The undulating voltage distribution for 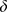-function input (Figure (2e)), reflects the nature of the ‘jump’ stochastic process. Heavier-tailed distributions generate faster transient responses to inputs and monotonically reach steady-state output firing rates. For example, the power-law distribution leads to an equilibrium firing rate 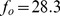 Hz and a transient time of 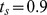 ms, while the Gaussian distribution results in 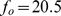 Hz and 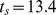 ms. Numerical results for all simulations are provided in Table (1). Even changing a small fraction of the synaptic weights can have a significant effect. For example, the 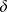-function distribution converges to the lowest steady state firing rate of 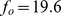 Hz (for computation of 95% confidence-intervals, see Table (1)). In contrast, the bimodal distribution which differs from the 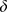-function by only 3.4% of synapses having a much higher weight, results in the highest steady state firing rate of 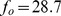 Hz. The tail of the synaptic weight distribution has an even larger effect on the transient times starting from rest - it is 16.2 ms for the 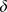-function, but only 2.6 ms for the bimodal distribution.

**Table 1:** Equilibrium rates and transient times.

	Mean input current matched (1 mV,1000 Hz)			Drift (1 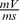 ) and diffusion (1.8 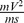 ) matched		
Distribution	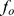 (Hz)	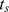 (ms)	95% CI Output rates	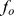 (Hz)	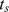 (ms)	95% CI Output rates
delta	19.6	16.2	(18.5,20.6)	22.0	11.0	(20.5,23.5)
Gaussian	20.5	13.4	(19.5,21.6)	21.8	10.6	(20.5,23.1)
exponential	21.3	10.6	(20.3,22.4)	21.5	10.2	(20.4,22.7)
lognormal	21.5	8.2	(20.4,22.6)	21.1	9.6	(20.1,22.1)
bimodal	28.7	2.6	(27.5,29.9)	15.9	4.2	(15.3,16.6)
power law	28.3	0.9	(27.0,29.6)	13.6	2.7	(13.3,13.9)

Table shows the equilibrium output firing rates 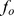, transient times 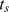 to 10% of 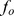 and 95% confidence intervals for 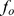 for matched synaptic weight distributions. 95% confidence intervals are calculated for 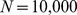 neurons with bin-size 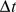 = 2 ms.

Since the network we study is a feed-forward network, the mean synaptic delay results in a simple time translation of the responses. However, the overshoot of steady state seen in Figure (2f) decreases as the variance in the distribution of synaptic delays increases (see Methods: Synaptic delays and Figure (S8)) relative to the membrane time constant 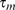.

To analyze what characteristic of the synaptic distribution is most important for the fast response observed for our heavy-tailed distributions, we generated more than 1000 random distributions matched to have the same mean synaptic weight and input rate (Methods: Tail weight numbers) and analyzed the responsiveness of a neuronal population (time to a fraction of the equilibrium firing rate) as a function of the moments of the synaptic distribution (Table (2) and top row of Figure (S10)). Higher order moments explain part of the variance observed in the responsiveness. However, a larger fraction of the variance can be explained by introducing a set of measures specifically designed to quantify the number of strong synapses (Table (2) and bottom row of Figure (S10); see also Figure (S9) for our six chosen distributions). These are the *tail weight numbers* 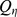, the density above threshold 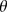 of 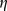 convolutions of the effective synaptic weight distribution:
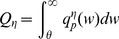
(6)where 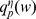 is related to the synaptic weight distribution 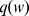 by,
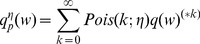
(7)and 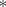 represents a convolution. 
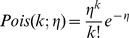
 represents a Poisson process with mean 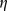 and 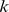 events occurring in a time-step. By definition, 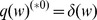, 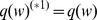, 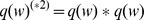 and so on. 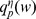 thus represents the average distribution of depolarization of a single neuron, when each neuron in the population receives 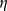 excitatory inputs on average. 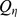 then represents the fraction of neurons that spike in a neuronal population starting at rest when each neuron in the population receives 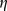 excitatory inputs on average.

**Table 2:** Explaining transient times with moments and tail weight numbers.

Moment	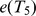	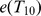	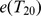	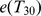
mom2	0.1739	0.1030	0.0391	**0.0165**
mom3	0.0479	**0.0177**	**0.0233**	0.0582
mom4	**0.0270**	0.0622	0.1580	0.2550
mom5	0.1179	0.2226	0.3874	0.5136

Table shows the sum of squared residuals 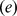 for best fit exponentials to 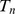 (the times taken to reach 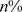 of the equilibrium firing rate), for the first few moments of 1222 randomly generated synaptic weight distributions between 0 and 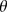. For both moments and tail weight numbers, the entries in bold in each column correspond to the lowest value of 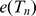. Tail weight numbers provide a better fit to transient times 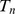 than moments.

### Input-output characteristics for different synaptic weight distributions

**Figure 3:**
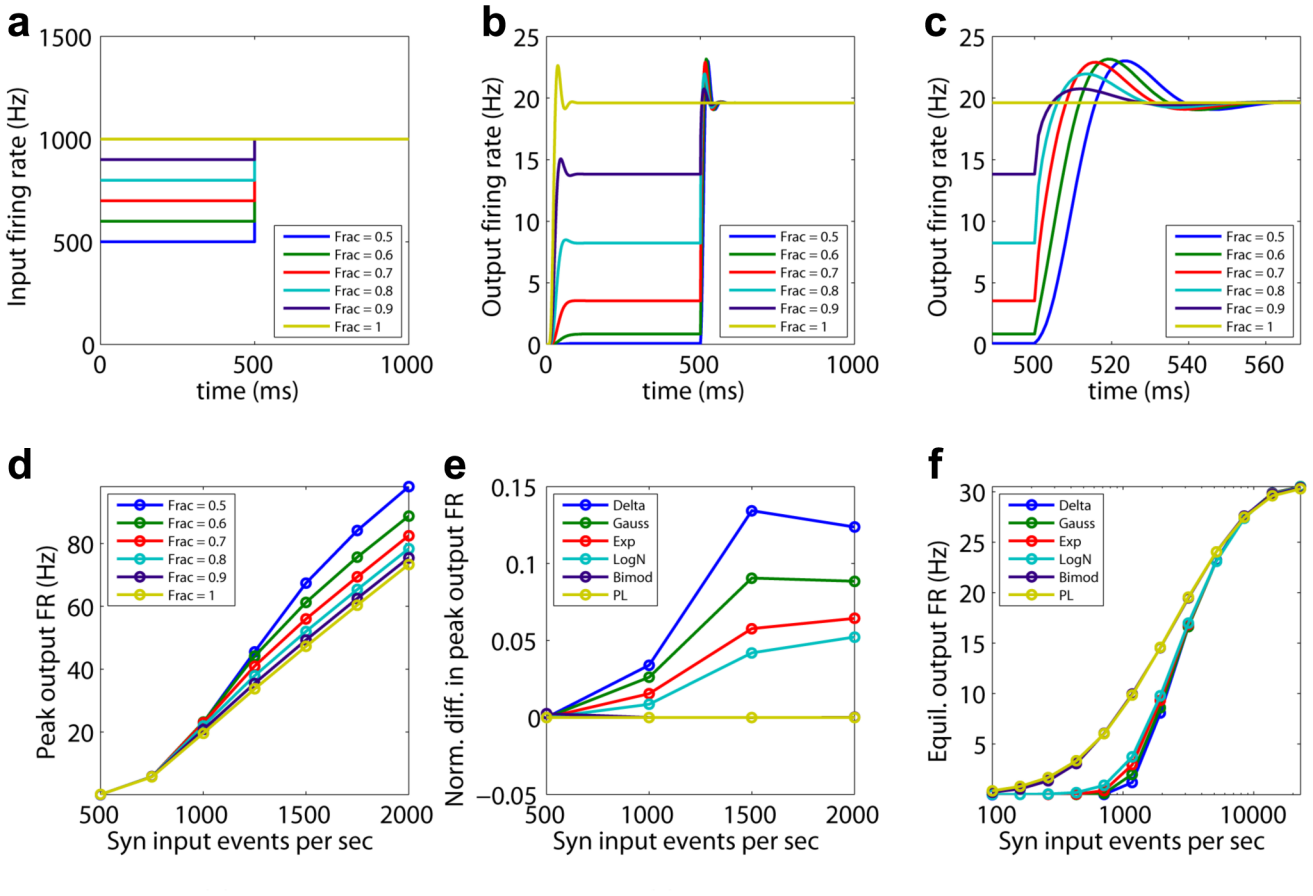
Input-output characteristics. Top row: Protocol used to investigate the response of the neuronal population with a given excitatory synaptic weight distribution to a sudden perturbation in its synaptic input. a) Input rate as a function of time. For the first 500 ms, the cumulative synaptic input rate is varied between 500 and 1,000 Hz, expressed as a fraction of the 1,000 Hz base input rate (see Methods: Input-Output curves). The population evolves according to equation (3) for a 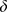-function distribution of synaptic weights. At 500 ms, the input firing rate instantaneously returns to the base rate of 1000 Hz. b) Output firing rate as a function of time for 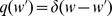. The peak output rate attained provides a measure of how strongly the system responds to sudden changes in its input rate. c) Zooming in onto the transient response in (b). The smaller the difference in input rate, the quicker the response of the network, with less overshoot. Bottom row: Quantifying response to sudden changes in input rate. d) Peak output firing rate for different fractions of the base input rate as a function of base input rate for 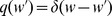. e) Difference in the peak output firing rate normalized by the difference between input rates, when the input is instantaneously changed from 1/2 the base rate to the full base rate. Normalized differences in output rates for different synaptic weight distributions are plotted as a function of the full input base rate. Heavier-tailed distributions result in lesser overshoot. f) Semi-log plot of the steady state output firing rate as a function of the input firing rate, for different synaptic distributions with the inclusion of short-term synaptic depression. Onset of saturation for very high effective synaptic input rates is evident. Note the greater response of the heavier-tailed power-law and bimodal distributions for lower input firing rates, leading to higher dynamical range.

Having established a measure of population activity when all neurons start at rest, we examined the dynamics resulting from an equilibrium different from rest (see Methods: Input-output curves). Mathematically, this amounts to analyzing the effect of synaptic weight distribution on the transient dynamics from one stationary solution 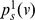 to another stationary solution 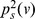, when the input synaptic event rate in equation (3) is instantaneously changed by a constant 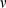 from 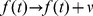. The different synaptic distributions result in an overshoot of the population's eventual equilibrium firing rate. The overshoot becomes smaller for heavier-tailed distributions. For the power-law, there is no overshoot present. The amount of overshoot is a measure of the stability of the neuronal population to sudden changes in input firing, given a distribution of synaptic weights. The weight of the tail in the synaptic distribution correlates with the stability of the system (Figure (3) and Figure (S12)).

The DiPDE formalism also enables insights into the influence of short-term synaptic depression (STSD), which is known to play a key role in neural network homeostasis and in the generation of multiple network states [41] via a Tsodyks-Markram mechanism [42]. With the inclusion of synaptic depression (see Methods: Implementation of short-term synaptic depression), the output firing rates for all six chosen synaptic weight distributions begin to saturate when the effective input synaptic events per second (integrated over all the synapses impinging onto a neuron) are around 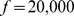 Hz (Figure (3f)). Heavier-tailed synaptic weight distributions lead to a higher dynamic range (Table (S4)).

### Effect of synaptic weight distribution on instantaneous response to external fluctuations

**Figure 4:**
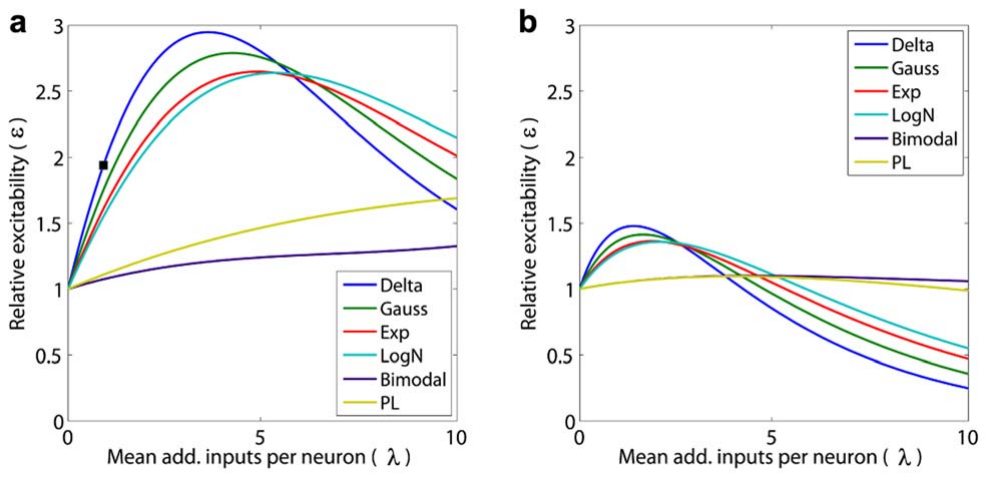
Effects of external fluctuations in synaptic input due to different excitatory synaptic weight distributions starting from the equilibrium of Figure (2) obtained with an input rate 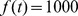 Hz. The graphs show the relative excitability 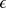 as a function of the additional synaptic input 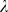 per neuron in the population on average (so that 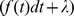 is the mean number of inputs per neuron on average in a time 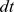), a) without and b) with synaptic depression. The black square indicates the relative excitability 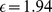 for a population with a 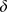-function distribution of synaptic weights with 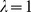 additional synaptic inputs per neuron on average. The corresponding relative excitability with a bimodal distribution is 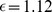. Heavy-tailed distributions lead to smaller changes in excitability due to fluctuations in synaptic input.

In the preceding analysis, we saw how perturbations of the input event rate in a population with different synaptic weight distributions affect the entire time-course of the population's evolution from an initial to a final equlibrium. In contrast, we also analyzed how fluctuations in inputs (see Methods: Fluctuation Analysis) affect the instantaneous change in equilibrium firing rate of neuronal populations with different synaptic weight distributions (Figure (4), Figure (S13) and Figure (S14)).

To quantify the instantaneous change in the equilibrium firing rate, we make use of a closely-related variant of the tail weight numbers defined in equation (6). Instead of starting with an initial probability distribution for the membrane potential corresponding to all neurons at rest, we use the stationary probability distribution 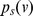 obtained from the solution to equation (5) with input rate 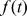 to define,
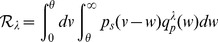
(8)where 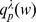 is as defined in equation (7). 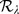 thus represents the fraction of neurons that spike in a neuronal population starting from the stationary distribution 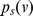, when each neuron in the population receives 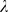 *additional* inputs on average. This means that, effectively in a time 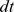, the total number of inputs becomes 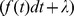.

Using 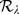, we can define the relative excitability 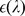 of a neuronal population with a given synaptic weight distribution as
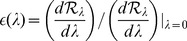
(9)This is a measure of susceptibility of the neuronal population. If 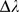 is the increase in input fluctuations for a population with 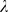 additional inputs per neuron on average, 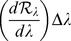 is the corresponding increase in the output firing rate of this population. A relative excitability 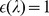 implies that the additional instantaneous response to an external input is independent of other inputs received at the same time. Starting from the equilibrium of Figure (2), for all synaptic weight distributions, the relative excitability initially rises (Figure (4a)). In this case, it can be partially compensated by synaptic depression (Figure (4b)). Table (S5) lists the maximum relative excitabilites for all the distributions, without and with synaptic depression. Results for an equilibrium obtained with a higher input firing rate have been presented in Figure (S14). Heavier-tailed distributions of synaptic weights cause smaller changes in relative excitability of the neuronal population.

### Strong-sparse and weak-dense synapses: An example

A few strong synapses can exert an undue large influence on the mean response of the population. Consider the unimodal 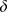-function synaptic distribution, whose weight is set at 1 mV and the bimodal distribution, where 96.6% of the synapses have 0.5 mV weight, while 3.4% of its synapses are much larger at 15 mV (an even more extreme case with the two distributions differing in only 0.1% of their synapses is presented in Figure (S11)). Even though both the average synaptic input and the total current are the same for both distributions, their steady-state firing rates are 19.6 and 28.7 Hz, their transient times are 16.2 and 2.6 ms and their dynamic ranges (see Table (S4)) are 10.22 and 31.83 respectively. More dramatic is the manner in which these two distributions react to synaptic fluctuations. When each neuron in the population receives 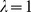 additional inputs on average the relative excitability for the 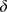-function distribution is 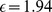, while that for the bimodal distribution is 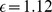 (see Figure (4a)).

## Discussion

Our study provides an advance on two fronts, computationally and neurobiologically. First, we developed and validated a semi-analytic method to model the sub-threshold membrane potential probability distribution and the firing rate of homogeneous neuronal populations with finite synaptic inputs. Second, we apply this method to explore the effect of varying synaptic weight distributions on equilibrium and transient population characteristics. From a methodological standpoint, the DiPDE formalism reproduces population behavior from aggregate simulations of identical point neurons, without the need to run the large-scale simulations themselves with the attendant computational costs (see Text (S3) and Table (S1)). The Fokker-Planck equation works best in the regime where the synaptic distribution and input firing rates approach a continuous process. It does not accurately model the response to even rare high-amplitude (‘jump’) synaptic inputs. By contrast, the ability of the DiPDE formalism to model both jump and continuous processes makes it a powerful framework for modeling single as well as multiple interacting neuronal populations.

This *population statistics* method makes possible the modeling and characterization of the membrane potential distribution and the spiking statistics of very large networks of distinct but homogeneous populations of hundreds of neuronal cell types in numerous brain regions. This would be relevant, for instance, when modeling the resting state activity at the cellular level throughout the awake or the sleeping brain. Second, in order to understand the biological significance of powerful but rare EPSPs [7], [18], [19], we used the DiPDE formalism to compare distributions which contain some very strong synapses against distributions with a preponderance of small EPSPs (but that are matched in their mean and variances). We will refer to the distributions as heavy-tailed, but a more quantitative description is presented in Methods: Tail weight numbers. We showed that power law and related heavy-tail distributions lead to faster transient behavior than non-heavy-tailed distributions (Figure (2f) and Figure (3f)). Second, heavy-tailed synaptic distributions lead to a higher dynamical range (Figure (3f)) than non-heavy-tailed distributions. Third, heavy-tailed distributions are much less sensitive to random fluctuations in synaptic activity (Figure (4)). All three properties associated with such heavy-tailed distributions can be functionally advantageous compared to matched synaptic input with no such large synapses. For example, faster transient responses would be desirable when a potential prey needs to detect the presence of a predator and escape. Higher dynamical ranges would be useful when sensory systems need to respond external stimuli over a large range, without the need to have multiple networks designed to respond over smaller ranges. The lesser sensitivity of heavy-tailed distributions to random fluctuations in synaptic activity can be useful when the response to a stimulus needs to be independent of its context.

Teramae and colleagues [17] recently published a joint computational and physiological investigation into the role of strong but sparse excitatory EPSPs, superimposed onto a very large pool of weak EPSPs and discovered the critical role that the former play in generating and sustaining long-term, low-frequency spontaneous firing activity in mixed excitatory-inhibitory neuronal networks in the absence of sustained external input or NMDA synapses. Such states allow the neurons to often reside one synaptic input away from the threshold 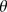, thereby leading to high correlation between the pre-synaptic and post-synaptic neurons of the strong synapses.

Such investigations highlight the need to focus the attention of electrophysiologists studying synaptic transmission *in vivo* onto the critical role that such rare, “Black Swan”-like, events can play in the day-to-day life of the brain.

Given the large biological and instrumental noise present in synaptic measurements, in particular under *in vivo* conditions, distinguishing between these distributions in practice would not be easy as it would require recordings of long duration to collect the relevant statistics. For experimental purposes, the numbers of independent recordings of sub-threshold steady state membrane potential required to differentiate between different synaptic weight distributions have been provided in Table (S2) and Table (S3). Yet as shown here, large but rare excitatory synaptic inputs can exert undue influence on population dynamics and robustness. These conclusions are very important for the emerging field of connectomics: a weighted graph description for neuronal networks in which each node represents a homogeneous population and each connection is characterized by the mean postsynaptic current is not sufficient. The network has to be either refined to individual neurons or expanded to include knowledge of the entire distribution of postsynaptic currents. The method further developed here facilitates the modeling and characterization of the membrane potential distribution and the spiking statistics of very large networks of distinct but homogeneous populations of hundreds of neuronal cell types in numerous brain regions.

## Methods

### Simplifications

A simplified case can be obtained if all EPSPs are assumed to have the same value 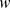; that is, if 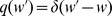. Equation (3) becomes:
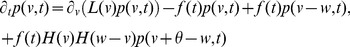
(10)This is a first order partial differential equation with displacement (DiPDE). For numerical solutions of equation (10), the matrix obtained by discretizing 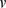 is as sparse as the matrix needed to solve the Fokker-Planck equation.

If 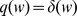, then equation (5) reduces to a delay differential equation (DDE)
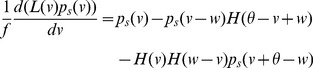
(11)


### Boundary conditions

In equation (3), if the additional depolarization after the neuron crossed threshold is neglected, the equation becomes
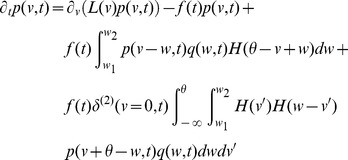
(12)where 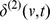 is the two-dimensional Dirac 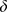-function and 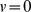 corresponds to the resting potential.

If 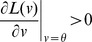, then there is an additional contribution 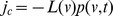 from the continuous processes to the probability flux through threshold. The output firing rate is now given by,
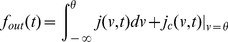
(13)


### Population simulations

Numerical simulations, against which the Fokker-Planck (for current-based synapses) and DiPDE formalisms were compared, were performed by invoking the NEST simulator after writing the code in PyNN.

For current-based synapses, simulations were performed for a population of 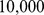 independent and identical leaky integrate-and-fire (LIF) neurons with decaying exponential post-synaptic current. With the resting and reset potential being equal and denoted by 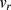, the neuron parameters were chosen to be 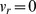 mV, 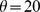 mV, membrane time constant 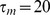 ms, membrane capacity 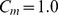 nF, refractory period 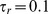 ms, input resistance 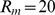 M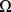 and decay time 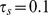 ms for excitatory synapses. All neurons in the population were supplied with a 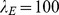 Hz Poisson excitatory input of amplitude 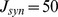 nA (although this is a huge current, with our choice of parameters, the effective charge deposited on the neuron is given by 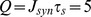 pC) and the membrane potential was recorded over a simulation time of 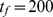 ms with a time-step of 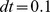 ms. The recorded voltages were then analyzed to obtain the probability distributions shown in Figure (1). The recorded spike-times were binned with an interval of 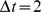 ms and the resultant output firing rate was obtained as shown in Figure (1d). For simulation results presented in Figure (S1), we used 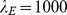 Hz Poisson excitatory input of amplitude 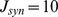 nA.

For conductance-based synapses, simulations were performed for a population of 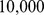 independent and identical leaky integrate-and-fire (LIF) neurons with decaying exponential post-synaptic conductance. The neuron parameters were chosen to be 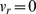 mV, 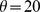 mV, excitatory reversal potential 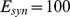 mV, membrane time constant 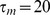 ms, membrane capacity 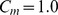 nF, refractory period 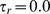 ms and decay time 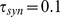 ms for excitatory synapses. All neurons in the population were excited by external Poisson input with input firing rate 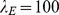 Hz and peak synaptic conductance 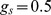 µS and their membrane potential was recorded over a simulation time of 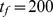 ms with a time-step of 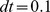 ms. The recorded voltages were then analyzed to obtain the probability distributions shown in Figure (S3). The recorded spike-times were binned with an interval of 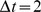 ms and the resultant output firing rate was obtained as shown in Figure (1g) and Figure (S3c).

Simulations were also performed for a population of 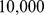 independent and identical exponential integrate-and-fire (EIF) neurons with conductance-based synapses without adaptation. The neuron parameters were chosen to be 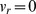 mV, 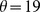 mV, 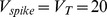 mV, slope-factor 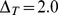 mV, excitatory reversal potential 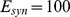 mV, membrane time constant 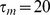 ms, membrane capacity 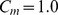 nF, refractory period 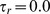 ms and decay time 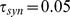 ms for excitatory synapses. The adaptation parameters were chosen to be 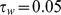 ms, and 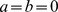, such that adaptation was absent. All neurons in the population were excited by external Poisson input with input firing rate 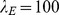 Hz and peak synaptic conductance 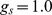 µS and their membrane potential was recorded over a simulation time of 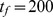 ms with a time-step of 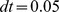 ms. The recorded spike-times were binned with an interval of 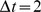 ms and the resultant output firing rate was obtained as shown in Figure (1h).

### Numerical solutions

To solve the evolution equation (3) for the probability density, we first solve the advection (leak) portion of the equation 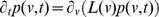, and then include the synaptic terms to calculate the overall time derivative. We then march the solution for 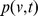 forward in time using an explicit first-order-in-time scheme with constant time step. Based on the general explicit scheme criterion 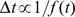, we use a sufficiently small time step to ensure stability. This scheme is conservative; the integral of the probability distribution is not affected by numerical errors.

For the standard LIF neuron with membrane time-constant 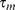, the leak term is linear with 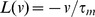. The ODE 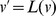 has an analytical solution and the method of characteristics provides the full solution for the advection term. In order to ensure an exact implementation of the leak term, we use a geometric binning scheme for the membrane potential, with the bin ratio determined by the product of leak 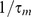 and time-step 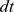. The bin-edges are determined, starting from threshold membrane potential 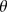, using
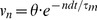
(14)A total of 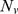 bin-edges are generated until the first bin between the resting potential 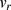 and 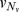 is at least as small or smaller than the first bin generated from 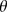. Mathematically, the lower bound on the number of bin-edges 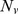 is given by the condition
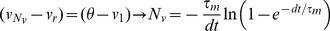
(15)since we have chosen 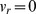 and the second equality follows from equation (14). Increasing the numbers of bins beyond this lower bound increases the accuracy, but also increases the computational cost involved (see Text (S3)).

For more general forms of the leak term (for e.g, the exponential integrate-and-fire (EIF) neuron; see Methods: Exponential integrate-and-fire neurons) the ODE 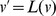 can be solved numerically starting from the threshold membrane potential in order to generate the bins.

At each time-step 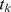, the probability distribution for membrane potential 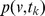 (which is a 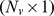 vector) is first evolved with the leak term and then the synaptic input. With our non-uniform binning scheme, the evolution of the probability distribution for the membrane potential due to the leak reduces to a single-index shift towards the resting potential and reduces the error due to numerical diffusion at each time step. To implement the effect of instantaneous synaptic input, including the effect of any excess input above the threshold 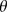 the input distribution of synaptic weights is first used to construct a 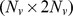 transition matrix 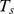. The 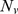 additional columns per row in 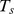 are used to keep track of the effect of excess synaptic input above 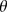. If this additional depolarization due to super-threshold inputs is ignored and the membrane potential is reset to zero upon exceeding 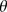, then 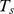 is just a 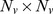 matrix. This is then used to construct an effective transition matrix
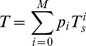
(16)where
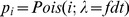
(17)represents the probability for the neuronal population to receive 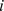 inputs in a time 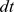 from an external homogeneous Poisson process with mean input rate 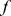. The Poisson process is truncated at a sufficiently high value of 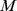 such that 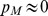. For inhomogeneous Poisson inputs, an effective transition matrix would have to computed at each time step 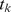, depending on the input rate 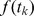. Each off-diagonal element in the effective transition matrix 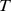 represents the proportion of neurons in the population receiving a specific synaptic input. The probability distribution 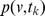 for the membrane potential at each time-step 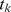 is then multiplied by this transition matrix 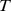 to generate the new probability distribution after synaptic input. The case for non-instantaneous synapses has been discussed below in Methods: Non-instantaneous synapses.

### Current-based synapses

In order to compare results with simulations, we need to match the total synaptic input to the neuronal population with that from simulations. The DiPDE formulation is exact when implementing instantaneous synapses 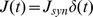.

The synaptic weight 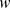 is equated with the maximum depolarization of membrane potential (peak EPSP) achieved after input from an exponentially decaying current-based synapse 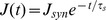,
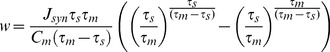
(18)This value is reached after a time
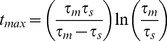
(19)


For our choice of simulation parameters outlined above 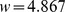 mV provides good agreement with simulations as shown in Figure (1). The time-step was chosen to be 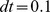 ms to match with simulations. Another constraint imposed by the nature of the numerical solution is that the discretization of the membrane potential means that it is impossible to have the initial probability distribution to be a sharp 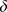-function at 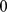 mV. The initial probability distribution is therefore spread uniformly over the width of the first voltage bin in both DiPDE and simulations.

### Expected 95% intervals for spike counts

The underlying stochastic process in equation (3) assumes Poisson-distributed inputs. If an equivalent numerical simulation with input firing rate 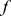 involves a finite population of 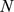 neurons and the resultant spikes are binned in 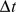 time intervals, then there are 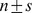 inputs per bin, with 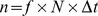 and 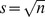. The relative variance in the number of inputs per bin is 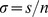.

Expected 95% intervals for output spike counts can then be obtained as follows. At each time step, the solution obtained from equation (3) for a given distribution of synaptic weights 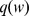 with a given input rate 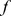 is additionally subjected to input rates 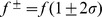 and the corresponding output rates 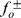 are calculated. These give the expected 95% intervals to the mean output firing rate 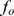. The 95% intervals for the expected spike counts per 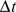 time-bin for a population 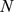 neurons are in the interval between 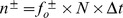.

Figure (1f) shows the output firing rates corresponding to the expected 95% intervals for results presented in Figure (1d). For a population of 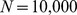 neurons and a bin size of 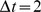 ms, the 95% interval for expected output spike counts per bin obtained from DiPDE is (90.3, 99.6) with corresponding output firing rates of 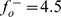 Hz and 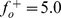 Hz respectively. For simulations, the 95% interval for spike counts corresponds to (90.5, 104.5).

### Non-instantaneous synapses

For non-instantaneous synapses, upper and lower bounds on the output firing rate can be obtained. The exponentially decaying current-based synapses used here in simulations (see (Materials and Methods: Current-based synapses)) result in an exact EPSP given by 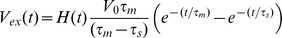 for synaptic input at time 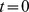. 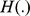 is the unit step function. If 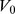 is the total charge deposited by an instantaneous, excitatory current-based synapse on a neuron with normalized capacitance at time 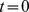, then the corresponding EPSP is given by 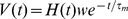, 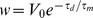 is the synaptic weight used in equation (3).

Setting 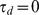 corresponds to equating the total charge, while setting 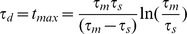 corresponds to equating the maximum depolarization. Figure (S4) shows the output firing rates obtained from DiPDE after equating the total charge, the maximum depolarization, an intermediate estimate obtained by setting 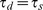 and that obtained by setting 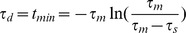, along with the corresponding numerical simulation.

A lower bound on the output firing rate can obtained by equating the maximum depolarization, 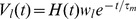 with 
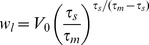
 corresponding to 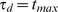. Equating the total charge 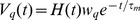 with 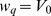 corresponding to 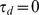 provides an estimate of the output firing rate. An upper bound can be obtained by taking 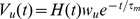 with 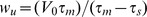 corresponding to 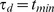. Figure (S5) shows a plot of the different EPSPs obtained with 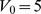 mV, 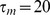 ms and 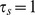 ms for synaptic input at time 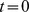.

For 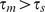, a formal proof for the upper and lower bounds can be provided as follows. Let 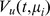, 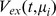 and 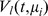 represent the EPSPs due to synaptic input at time 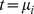. The maximum depolarization attained due to 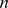 inputs can be represented as:
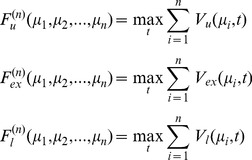
(20)For any 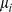, 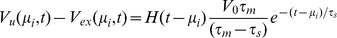. Therefore, 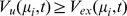 for all times after the synaptic input, so that 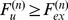. Hence, using 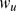 in equation (3) an upper bound for the output firing rate is obtained.

For the lower bound, consider the difference between 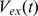 and 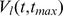. For all 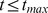, since 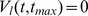, 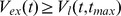. For 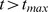, 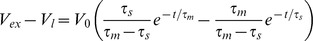. Using this, it is straightforward to show that 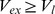 when 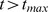. Thus 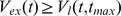 for all times 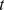 and hence 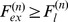. The time translation by 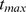 does not affect the result since 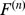
's have been defined to be the maximum depolarization over all times 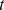.

### Conductance-based synapses

For current-based synapses, the change in membrane potential resulting from synaptic input is independent of the initial membrane potential. For a conductance-based synapse however, this change is proportional to the difference between the initial membrane potential and the reversal potential 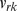 for the 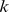 channels present in a particular synapse. Equation (3) becomes,
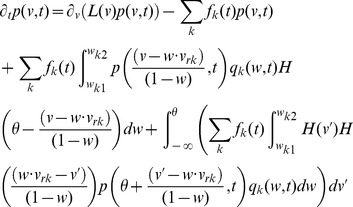
(21)


Because of the additional dependence on the membrane voltage and synaptic reversal potential 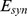, modeling an instantaneous change in voltage through a conductance-based synapse is not as straightforward as with a current-based synapse. To model this type of synapse efficiently, we introduce the non-dimensional quantity 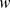 as in equation (21) above, which represents the instantaneous voltage change due to synaptic activation as a fraction of the charge needed to reach the reversal potential from a given membrane potential. For example, 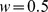 indicates that a single synaptic event would shift the neuron from its current membrane potential to halfway between the current voltage and the synaptic reversal potential. Mathematically, if 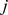 synaptic events have already occurred, then the change in membrane potential induced by the 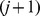-th synaptic input can be represented by,
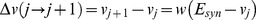
(22)For 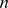 such synaptic events, it can be verified that this reduces to:
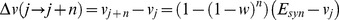
(23)This non-dimensionalization of synaptic weight for conductance-based synapses thus automatically rescales all weights to lie between zero and 1, and provides a simple way to model these synapses as generating instantaneous changes in membrane voltage.

For our choice of simulation parameters, the maximum depolarization achieved by a neuron starting from rest due to synaptic input with 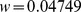 and 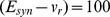 mV is 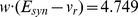 mV. This is obtained from the numerical solution of the equation for a LIF neuron with exponentially decaying conductance-based synapses (with time-step chosen to be 0.1 ms to match with simulations).

Figure (S3) shows that numerical simulations (see Methods: Simulations for parameters used in simulations) are in good agreement with numerical solutions of DiPDE for conductance-based synapses. The equilibrium output firing rate is 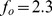 Hz and the transient time to firing is 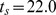 ms. For 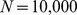 neurons and a bin size of 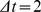 ms, the 95% confidence interval for the spike counts per bin is (43.0,47.6) corresponding to output firing rates of 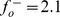 Hz and 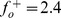 Hz.

For multiple neuronal populations connected to each other, one can generalize equation (21) to a system of equations,
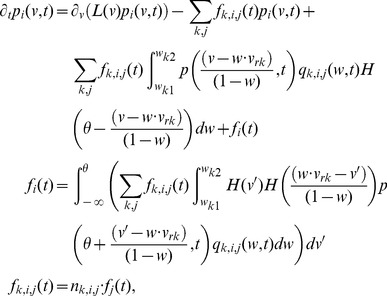
(24)while defining two synaptic weight matrices,

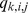
, which represents the probability density of the strength of synapses of type 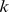, from population 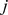 to population 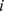,

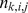
, which represents the probability density of number of release sites at synapses of type 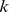, from population 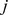 to population 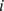.

### Exponential integrate-and-fire neurons

The DiPDE formalism (equation 3) can be generalized to other types of neurons such as for e.g - the exponential integrate-and-fire (EIF) neuron. The membrane potential dynamics for the EIF neuron with excitatory conductance-based synapses is governed by
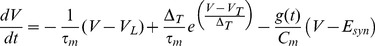
(25)where the first two terms on the RHS contribute to the leak and the third term corresponds to the synaptic input. 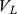 represents the leak reversal potential, 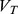 is the spike detection threshold, 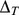 is the slope-factor, 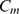 is the membrane capacitance, 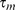 is the membrane time-constant and 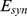 is the excitatory reversal potential. In the absence of synaptic input, the leak portion can be solved numerically to obtain the discretized non-uniform bins for the membrane potential.

For the exponential integrate-and-fire (EIF) neuron, the numerical solution of the equation with exponentially decaying conductance-based synapses gives 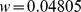. With 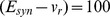 mV, this means that the maximum depolarization achieved by a neuron starting from rest is 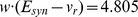 mV (with time-step chosen to be 0.05 ms to match with simulations).

Figure (1h) shows that the output firing rate obtained from numerical simulations (see Methods: Simulations for parameters used in simulations) is in good agreement with that obtained from the numerical solution of DiPDE. The equilibrium output firing rate is 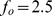 Hz and the transient time to firing is 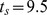 ms. For 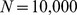 neurons and a bin size of 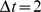 ms, the 95% confidence interval for the spike counts per bin is (48.5,53.1) corresponding to output firing rates of 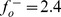 Hz and 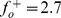 Hz.

### Numerical solution of Fokker-Planck equation

The Fokker-Planck equation for current-based synapses was solved using an explicit Forward-Time Centered-Space (FTCS) scheme. As in the DiPDE, the leak term was solved analytically, although the voltage discretization was kept uniform in order to be compatible with the standard Centered-Space discretization used for the diffusion term. To ensure that the discretization was sufficiently fine (especially since FTCS is only first-order accurate), we compared the equilibrium firing rate generated by this scheme against the analytical solution [43] given by
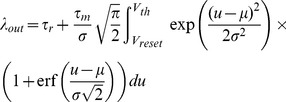
(26)where 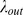 is the analytical firing rate, 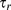 is the refractory period and 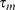 is the membrane time constant. 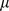 and 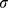 are parameters related to the drift and diffusion coefficients in the Fokker-Planck equation, and are given by
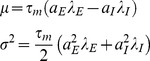
(27)where 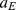 and 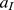 are excitatory and inhibitory synaptic weights respectively, while 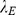 and 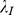 are the corresponding input firing rates.

### Matched synaptic distributions

The effect of various matched synaptic weight distributions on the population dynamics in feed-forward networks was investigated using the DiPDE formalism. The top panel of Figure (2) showed that two self-similar Gaussian distributions matched to produce the same average synaptic current resulted in different steady-state output firing rates and sub-threshold membrane potential distributions. In the regime when the firing is primarily driven by drift, the difference in output firing rates is small as shown in the top panel of Figure (S6). The higher-mean Gaussians result in an steady state output firing rate of 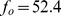 Hz and 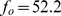 Hz, while the lower mean Gaussians lead to 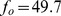 Hz and 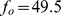 Hz.

If the firing is driven exclusively by variations in input (Gaussian distributions with balanced excitation and inhibition), the differences in output firing rates are large as shown in the bottom panel of Figure (S6). For the Gaussians with 6 mV mean and either 1 mV or 2 mV standard deviations, the steady state output firing rates are 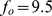 Hz and 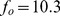 Hz respectively. The steady state output firing rates for the Gaussians with 3 mV mean and either 0.5 mV or 1 mV standard deviations are 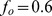 Hz and 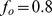 Hz respectively.

These results imply that population response is determined not only by the total current, but also by the mean synaptic weights.

For the bottom row of Figure (2), six different synaptic weight distributions were tuned to have the same mean (1 mV). Using the same input rate (1,000 Hz) for the all the distributions, the average synaptic current was the same. The corresponding expressions for these distributions normalized between 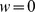 and 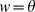 are:

- Delta : 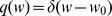 with 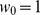 mV.
- Gaussian : 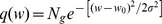 with 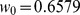 mV and 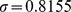 mV.
- Exponential : 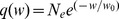 with 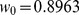 mV.
- Lognormal : 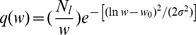 with 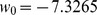 and 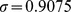.
- Bimodal : 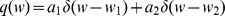 with 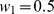 mV, 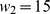 mV, 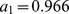 and 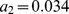.
- Power-law : 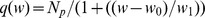 with 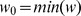 and 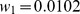 mV.

The 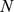
's represent the normalization constants. The corresponding zero-centered second moments are: Delta(1.0 mV^2^), Gaussian (1.3 mV^2^), Exponential (1.8 mV^2^), Lognormal (2.2 mV^2^), Bimodal (8.0 mV^2^) and Power-law (8.7 mV^2^). The firing rates and transient times corresponding to distributions for which the mean input current was matched (Figure (2) is provided in Table (1).

An alternate way to match synaptic weight distributions is to match the drift and diffusion, corresponding to what the mean and variance of the membrane potential would be if the neuron did not have a threshold, while allowing the input rates to vary. In the Fokker-Planck formalism, such distributions matched for drift and diffusion would have led to the same results.

Figure (S7) shows the differences in output firing rates and equilibrium membrane potential distributions obtained from eq. (3) for synaptic weight distributions matched for drift and diffusion. The input rates are: Delta (555.5 Hz), Gaussian (745.98 Hz), Exponential (952.38 Hz), Lognormal (1,062.15 Hz), inverse power-law (4,166.72 Hz) and Bimodal (1,825.33 Hz). The corresponding expressions for these distributions normalized between 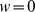 and 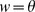 are:

- Delta : 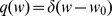 with 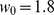 mV.
- Gaussian : 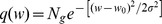 with 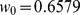 mV and 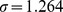 mV.
- Exponential : 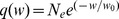 with 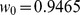 mV.
- Lognormal : 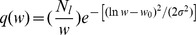 with 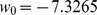 and 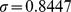.
- Bimodal : 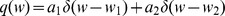 with 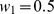 mV, 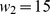 mV, 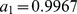 and 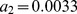.
- Power-law : 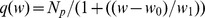 with 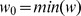 and 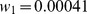 mV.

The 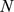
's represent the normalization constants.

The heavier-tailed distributions still lead to faster transients, however the steady-state firing rates now decrease with increasing heaviness of the tail in the distributions. This is in contrast to the case when the input currents were matched (Figure (2).

In order of increasing heaviness of the tail distributions, the equilibrium output firing rates and the transient firing times for the different distributions are provided in Table (1).

### Synaptic delays

We also implement synaptic delays within the DiPDE formalism. This is done by a using a queue to store the output firing rate, which is then accessed and updated depending on the distribution of synaptic delays. Figure (S8) shows the effect of synaptic delays on the output firing rate of a feed-forward network with different synaptic weight distributions in the presence of 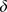-function and Gaussian distributions of synaptic delays with the same mean delay. Since the network is feed-forward, the mean delay simply translates the responses in time. Higher variance in the distribution of delays relative to the membrane time constant 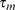 leads to lesser overshoot of the steady state.

### Tail weight numbers

The tail-heaviness of the synaptic weight distribution can be characterized by either constructing the moments of the distribution or a set of *tail weight numbers*. These numbers 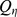 were defined in equations (6) and (7) and represent the fraction of neurons that spike in a neuronal population starting at rest when each neuron in the population receives 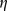 excitatory inputs on average. While higher order moments of the synaptic weight distributions explain part of the variance observed in the responsiveness, a larger fraction of the variance can be explained by the tail weight numbers.

Figure (S9) shows the values of the tail weight numbers 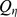 as a function of 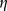 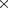 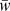 (the average synaptic weight) for distributions matched for mean input current (top left) and for drift and diffusion (top right). These numbers start increasing much quicker for heavier-tailed distributions as is evident from the lower panel of Figure (S9).

To test which key characteristic of synaptic weight distributions better describes the transient times, we generated 1222 random distributions between 0 and 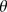, each with a mean of 2 mV. With an input firing rate of 500 Hz, we computed the output firing rates obtained from DiPDE and the corresponding transient times taken to reach different fractions of the steady state firing rate (time taken to reach 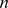
% of equilibrium firing rate denoted as 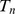 here) for all these matched distributions. Figure (S10) shows a scatter plot of 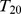 as a function of the first few moments and response numbers respectively for all these distributions along with the best-fit exponential curves (shown in red). Scatter plots as a function of tail weight numbers provide a better fit to the transient times than the moments. This can be seen from Table (2), which lists the sum of squared errors of the residuals 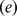 for each of the best fit exponentials for different 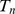
's as a function of the first few moments and tail weight numbers respectively. Plots for the other 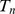 look similar to the plots for 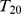 shown in Figure (S10).

### Input-output curves

Input-output curves in Figure (3) and Figure (S12) were generated using the following protocol: for a given fraction 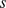 and base input rate 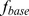, the DiPDE was solved first with an input firing rate 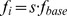. After 500 ms (at which time all runs had reached an equilibrium voltage distribution), the input rate was instantaneously changed to 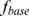. The resulting output firing rate 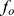 was calculated as the maximum output firing rate achieved after the instantaneous input rate change.

We quantified the spread in the input-output relationship due to different values of the fraction 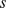 for a given base input rate 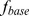 by measuring the slope, defined as follows:
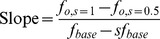
(28)


### Implementation of short-term synaptic depression

The most comprehensive treatment of synaptic utility would involve a single 3-D integro-differential equation where the probability distribution 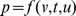 depends additionally on the synaptic utility. However, since this formalism is computationally more expensive and given that the two separate 2-D integro-partial differential equations provide adequate results in cases where the full joint probability can be expressed as a product of the marginals over the voltage and synaptic utility, we opted to model synaptic utility as a separate differential equation via a Tsodyks-Markram mechanism [42]. This additional equation describes the probability of a synapse to have a particular utility. The synaptic utility ranges from 0 to 1, with 1 indicating maximum availability of synaptic vesicles. Its recovery is governed by
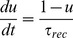
(29)In the pre-synaptic population, for all probability at the membrane potential threshold 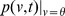, the synaptic utility is decremented using,
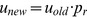
(30)where 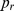 thus represents the fraction of synaptic vesicles available after a spike. For our analysis, we use 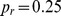 and 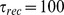 ms respectively.

Given the exponential form of equation (29), the synaptic utility axis (ranging from 0 to 1) is also discretized geometrically, similar to the voltage discretization for linear leak (equation (14)). Thus, at each time step, the pre-synaptic probability matrix undergoes a single-index shift towards full synaptic utility (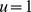).

When a non-zero proportion of the probability for a given pre-synaptic neuronal population is at threshold (i.e. there is spiking in the population), the output synaptic weight distribution from that population to the target population is convolved with the synaptic utility distribution,
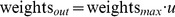
(31)and the utilities are subsequently modified according to equation (30).

### Fluctuation analysis

To analyze the effect of fluctuations generated by different synaptic distributions, the DiPDE numerical solution was evolved with a given synaptic distribution for 300 ms, long enough for the voltage distribution to reach equilibrium. At this point, to simulate fluctuations which occur on timescales 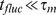, 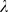 extra synaptic inputs are added per neuron on average to the population in a single time step. This is done by convolving the stationary probability distribution 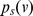 obtained from a given external input rate 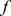 with the corresponding synaptic weight distribution 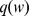 and the instantaneous change from the equilibrium firing rate is quantified using equation (9).

This same analysis was then conducted with synaptic depression during the fluctuation stage, starting with the stationary distribution obtained without synaptic depression. Unlike in the no-depression case, the synaptic distribution was scaled by the synaptic utility (using the method outlined in (Methods: Implementation of short-term synaptic depression)) after each additional synaptic event. The utility did not recover (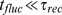). The utility fraction 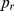, which represents the decrement in pre-synaptic utility after an input (equation (30)), was calculated for each synaptic distribution separately. Denoting the peak of the relative excitability curve generated from the no-depression case by 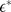 after 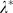 additional inputs per neuron on average, we chose the decrement 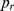 to be:
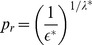
(32)Intuitively, with this choice, after 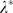 additional inputs per neuron on average, the relative excitability in the presence of synaptic depression gets closer to unity.

All the results for DiPDE simulations were obtained using MATLAB. The entire code base is available for download at http://download.alleninstitute.org/publications/the_influence_of_synaptic_weight_distribution_on_neuronal_population_dynamics/.

## Supporting Information

Figure S1Comparisons for excitatory, high-frequency and low-amplitude current-based synapses. Top panels show time evolution of the probability distribution of membrane potentials in the neuronal population obtained from a) simulations of 10,000 leaky integrate-and-fire neurons, b) the numerical solution to equation (3), and c) Fokker-Planck equation respectively. Bottom panels: d) Output firing rates as a function of time. e) The distribution of sub-threshold steady-state membrane potential after 200 ms. Poisson input for 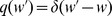 with synaptic weight (maximum EPSP) 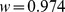 mV and input firing rate 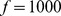 Hz.(EPS)

Figure S2Solutions to eq. (3) do not depend on the choice of time-step used in the numerical solution. Figure shows output firing rates for excitatory, current-based synapses as in Figure (1), obtained from numerical solutions of eq. (3) for different discretization time-steps with Poisson input for 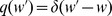 with synaptic weight (maximum EPSP) 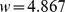 mV and input rate 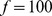 Hz. Choice of time-step does not affect numerical results with 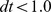 ms. Both the transient and steady-state firing rates presented in Figure (1) remain unchanged for different choices of time-steps in the numerical solution.(EPS)

Figure S3Comparisons for excitatory conductance-based synapses. Top panels show time evolution of the probability distribution of membrane potentials in the neuronal population obtained from a) simulations, and b) numerical solution to equation (21) respectively. Bottom panels: c) Output firing rates as a function of time. d) The distribution of sub-threshold steady state membrane potential. Poisson input for 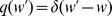 with maximum depolarization achieved by a neuron starting from rest 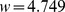 mV and input rate 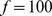 Hz.(EPS)

Figure S4For non-instantaneous synapses, estimates of the output firing rate can be obtained by using instantaneous synapses equated for the total charge (with normalized capacitance) or the maximum depolarization respectively. These are controlled by an additional parameter 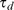 (see Methods: Non-instantaneous synapses). 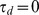 corresponds to equating total charge (green curve) and 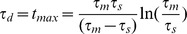 corresponds to equating maximum depolarization (red curve). An intermediate estimate for the output firing rate can be obtained if 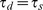 (light-blue curve). An upper bound can be obtained if 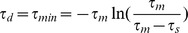 (purple curve). Simulations are for 10,000 LIF neurons with synaptic time-constant 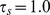 ms (dark-blue curve). Poisson input with 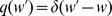, input rate 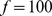 Hz, 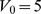 mV and 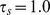 ms.(EPS)

Figure S5EPSPs from instantaneous current-based, excitatory synapses used for obtaining estimates of the output firing rate for non-instantaneous synapses (see Methods: Non-instantaneous synapses). 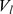 (red) is the EPSP obtained by equating the maximum depolarization, 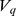 (dark blue) is the EPSP obtained by equating the total charge and 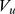
(light blue) is the EPSP used to obtain an upper bound on the output firing rate. 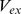 represents the exact EPSP for the non-instantaneous synapse.(EPS)

Figure S6Gaussian distributions of instantaneous synaptic weights with the same mean input current. Top Panels: a) Four Gaussian synaptic weight distributions. b) The output firing rates as a function of time when the four Gaussian synaptic inputs are activated with an input firing rate adjusted such that the mean input currents are equal. Both low-amplitude distributions (red and light-blue curves) have twice the input rate of the high-amplitude ones (dark-blue and green curves). In the absence of a threshold, the synaptic input would depolarize 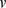 by 30 mV for the all the distributions. As we use a threshold of 20 mV, all the results are primarily driven by the mean input. In b), colors of curves correspond to the weight distributions shown in a). Output firing rates do not differ much when primarily driven by the mean input. Bottom Panels: c) Four synaptic weight distributions with Gaussian excitatory and inhibitory weights resulting in balanced excitation and inhibition. The population response is driven exclusively by variations in synaptic input. Input rate for all the distributions is 500 Hz. d) Output firing rates as a function of time. Results imply that population response is determined not only by the total current, but also by the variance of synaptic weights.(EPS)

Figure S7Distributions of synaptic weights with same mean input current and variance of membrane potential. a) Semi-log plot of different synaptic distributions, matched for drift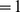 mV/ms and diffusion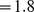 mV^2^/ms. b) Steady state sub-threshold membrane potential distributions. c) Output firing rates. Heavier-tailed distributions still produce quicker transients, but result in lower steady state output firing rates in contrast to (Figure (2)).(EPS)

Figure S8Effect of 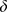-function and Gaussian distributions of synaptic delays on the overshoot of output firing rates of the population. All distributions have the same mean 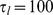 ms. Even in the presence of different distributions of synaptic delays, heavier-tailed distributions still lead to quicker transient responses as seen earlier in Figure (2). Variance in the distribution of delays affects the overshoot of equilibrium firing rate, with higher variances leading to lower overshoot.The top panels all correspond to distributions with the same mean synaptic weight (1 mV) and input rate (1000 Hz), while the bottom panels all correspond to distributions with mean synaptic weight (1 mV) and input rate (3000 Hz). For both rows from left to right, the first column shows the output firing rates with standard deviation 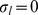 ms (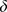-function distribution of delays), the second a Gaussian distribution with 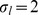 ms, the third a Gaussian distribution with 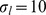 ms and, the last a Gaussian again with 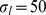 ms. The 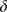-function delays just translate the responses for all distributions by a constant 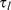. Higher variance in the distribution of delays leads to lower overshoot as can be seen going from left to right along each row. To obtain an appreciable decrease in the overshoot of the output firing rate, the delays have to be distributed with substantial standard deviation 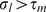, where 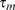 is the membrane time constant.(EPS)

Figure S9Top Panels: Tail weight numbers 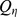 for synaptic weight distributions matched for a) mean input current and b) drift and diffusion (see Methods: Matched synaptic distributions). These are plotted as a function of 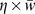 where 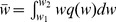 represents the average synaptic weight. Lower Panels: c) and d) Zooming in on top panel figures for low values of 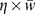. Heavier-tailed distributions have higher tail weight numbers for smaller values of 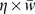.(EPS)

Figure S10Time taken to reach 20% of steady-state firing rate (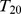) as a function of the first few moments (Top row: Panels (a), (b), (c), (d)) and tail weight numbers (Bottom row: Panels (e), (f), (g), (h)) for 1222 randomly generated synaptic weight distributions between 0 and 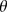 with mean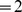 mV and input rate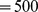 Hz. Exponential best fit curve shown in red.(EPS)

Figure S11Even 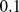
% of all synapses being strong makes a difference in the speed of the response, leading to a quicker transient time. Figure shows output firing rates for mean and drift-matched 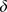-function of synaptic weights with 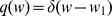 and bimodal distribution of synaptic weights with 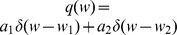. The 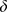-function has 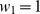 mV while the bimodal distribution has 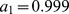 with 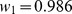 mV and 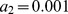 with 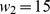 mV. The red arrow shows that already after a short time 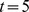 ms, the output rate for the 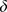-function distribution is 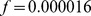 Hz while the output rate for the bimodal distribution is 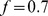 Hz.(EPS)

Figure S12Quantifying response to sudden changes in input rate for different synaptic weight distributions matched for mean input current. Peak output firing rates for different fractions of base input rate as a function of base input rate for a) 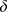-function, b) Gaussian, c) Exponential, d) Lognormal, e) Bimodal, and f) Power-law distribution of synaptic weights. Heavier-tailed distributions result in lesser overshoot.(EPS)

Figure S13Effect of external fluctuations on sub-threshold membrane potential distribution and excitability for different synaptic weight distributions, without and with synaptic depression. Simulations start from the equilibrium of Figure (2) and up to 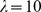 additional inputs per neuron on average are activated. From top to bottom, the rows represent results for the 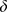-function, Gaussian, exponential, lognormal, power-law and bimodal distributions (Methods: Matched distributions) respectively. For each row, panels a) and b) represent the membrane potential distribution without and with synaptic depression, respectively. The membrane potential distribution at the initial equilibrium is represented in blue, following increment of 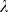 in steps of 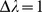 in green, and the final distribution is in red. For each row, panel c) shows the relative excitabilities, without and with synaptic depression.(EPS)

Figure S14Effects of fluctuations in synaptic input, starting from the equilibrium obtained for different synaptic weight distributions with mean = 1 mV and input rate 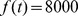 Hz. The figures show how the relative excitability 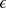 varies as a function of the additional synaptic inputs 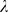 per neuron on average in the population (so that 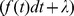 is the mean number of inputs per neuron on average in a time 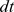), a) without and b) with synaptic depression. Starting from this higher input rate, relative excitability decreases for all synaptic weight distributions.(EPS)

Table S1Computational times. Table shows the simulation times in (s) with NEST and DiPDE for different choices of time step dt. The middle four columns correspond to simulation times with different numbers of neurons 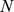 used in the NEST simulations. The last column shows simulation times for DiPDE with numbers in parantheses representing the number of non-zero elements 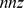 for a given time step 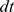.(PDF)

Table S2 Differentiating synaptic weight distributions matched for mean input current. Table shows the number of independent recordings of sub-threshold steady state membrane potential required to differentiate between synaptic distributions matched for mean input current. The values above the diagonal are the sample sizes needed for p = 0.01, and below the diagonal are the sizes for p = 0.05.
(PDF)

Table S3 Differentiating synaptic weight distributions matched for drift and diffusion. Table shows the number of independent recordings of sub-threshold steady state membrane potential required to differentiate between synaptic distributions matched for drift and diffusion. The values above the diagonal are the sample sizes needed for p = 0.01, and below the diagonal are the sizes for p = 0.05.
(PDF)

Table S4 Dynamical ranges. Table shows the upper (90%) and lower (10%) limits of steady state output firing rates and dynamical ranges (ratio of upper and lower steady state output firing rates) for synaptic weight distributions (as in Figure (2)) matched for mean input synaptic current. Dynamical range increases as the distributions get heavier-tailed.
(PDF)

Table S5 Relative excitabilities. Table shows the maximum relative excitability for synaptic weight distributions matched for mean input current, without and with synaptic depression. Heavier-tailed distributions lead to smaller changes in relative excitability.
(PDF)

Text S1 A brief review of stochastic processes focusing on elements needed for this study.
(PDF)

Text S2 Comparison of DiPDE and Fokker-Planck formalisms.
(PDF)

Text S3 Computational complexity with DiPDE.
(PDF)

Text S4 p-values for differences between sub-threshold steady-state membrane potential distributions obtained from different synaptic weight distributions.
(PDF)

## References

[1] Song S, Sjöström PJ, Reigl M, Nelson S, Chklovskii DB (2005) Highly Nonrandom Features of Synaptic Connectivity in Local Cortical Circuits. PLoS Biol 3: e68. 15737062 10.1371/journal.pbio.0030068 PMC1054880

[2] Sjöström PJ, Turrigiano GG, Nelson SB (2001) Rate, Timing, and Cooperativity Jointly Determine Cortical Synaptic Plasticity. Neuron 32: 1149–1164. 11754844 10.1016/s0896-6273(01)00542-6

[3] Mason A, Nicoll A, Stratford K (1991) Synaptic transmission between individual pyramidal neurons of the rat visual cortex in vitro. The Journal of Neuroscience 11: 72–84. 1846012 10.1523/JNEUROSCI.11-01-00072.1991 PMC6575189

[4] Lefort S, Tomm C, Floyd Sarria JC, Petersen CCH (2009) The excitatory neuronal network of the C2 barrel column in mouse primary somatosensory cortex. Neuron 61: 301–316. 19186171 10.1016/j.neuron.2008.12.020

[5] Frick A, Feldmeyer D, Helmstaedter M, Sakmann B (2008) Monosynaptic Connections between Pairs of L5A Pyramidal Neurons in Columns of Juvenile Rat Somatosensory Cortex. Cerebral Cortex 18: 397–406. 17548800 10.1093/cercor/bhm074

[6] Markram H, Lübke J, Frotscher M, Roth A, Sakmann B (1997) Physiology and anatomy of synaptic connections between thick tufted pyramidal neurones in the developing rat neocortex. The Journal of Physiology 500: 409–440. 9147328 10.1113/jphysiol.1997.sp022031 PMC1159394

[7] Ali AB, Deuchars J, Pawelzik H, Thomson AM (1998) Ca1 pyramidal to basket and bistratified cell epsps: dual intracellular recordings in rat hippocampal slices. The Journal of Physiology 507: 201–217. 9490840 10.1111/j.1469-7793.1998.201bu.x PMC2230771

[8] Sayer R, Friedlander M, Redman S (1990) The time course and amplitude of EPSPs evoked at synapses between pairs of CA3/CA1 neurons in the hippocampal slice. The Journal of Neuroscience 10: 826–836. 2319304 10.1523/JNEUROSCI.10-03-00826.1990 PMC6570142

[9] Feldmeyer D, Egger V, Lübke J, Sakmann B (1999) Reliable synaptic connections between pairs of excitatory layer 4 neurones within a single barrel of developing rat somatosensory cortex. The Journal of Physiology 521: 169–190. 10562343 10.1111/j.1469-7793.1999.00169.x PMC2269646

[10] Isope P, Barbour B (2002) Properties of Unitary Granule CellPurkinje Cell Synapses in Adult Rat Cerebellar Slices. The Journal of Neuroscience 22: 9668–9678. 12427822 10.1523/JNEUROSCI.22-22-09668.2002 PMC6757845

[11] Brunel N, Hakim V, Isope P, Nadal JP, Barbour B (2004) Optimal Information Storage and the Distribution of Synaptic Weights: Perceptron versus Purkinje Cell. Neuron 43: 745–757. 15339654 10.1016/j.neuron.2004.08.023

[12] Ikegaya Y, Sasaki T, Ishikawa D, Honma N, Tao K, et al (2012) Interpyramid Spike Transmission Stabilizes the Sparseness of Recurrent Network Activity. Cereb Cortex 23 ((2)): 293–304. 22314044 10.1093/cercor/bhs006

[13] Miles R (1990) Variation in strength of inhibitory synapses in the CA3 region of guinea-pig hippocampus in vitro. The Journal of Physiology 431: 659–676. 1983123 10.1113/jphysiol.1990.sp018353 PMC1181797

[14] Holmgren C, Harkany T, Svennenfors B, Zilberter Y (2003) Pyramidal cell communication within local networks in layer 2/3 of rat neocortex. The Journal of Physiology 551: 139–153. 12813147 10.1113/jphysiol.2003.044784 PMC2343144

[15] Softky WR (1995) Simple codes versus efficient codes. Current Opinion in Neurobiology 5: 239–247. 7620313 10.1016/0959-4388(95)80032-8

[16] Valiant LG (1994) Circuits of the mind. New York: Oxford University Press.

[17] Teramae JN, Tsubo Y, Fukai T (2012) Optimal spike-based communication in excitable networks with strong-sparse and weak-dense links. Sci Rep 2: 485. 22761993 10.1038/srep00485 PMC3387577

[18] Molnár G, Oláh S, Komlósi G, Füle M, Szabadics J, et al (2008) Complex events initiated by individual spikes in the human cerebral cortex. PLoS Biol 6: e222. 18767905 10.1371/journal.pbio.0060222 PMC2528052

[19] Komlósi G, Molnár G, Rózsa M, Oláh S, Barzó P, et al (2012) Fluoxetine (prozac) and serotonin act on excitatory synaptic transmission to suppress single layer 2/3 pyramidal neuron-triggered cell assemblies in the human prefrontal cortex. The Journal of Neuroscience 32: 16369–16378. 23152619 10.1523/JNEUROSCI.2618-12.2012 PMC3752144

[20] Van Kampen N (2007) Stochastic Processes in Physics and Chemistry. North-Holland Personal Library. Elsevier. URL http://books.google.com/books?id=N6II-6HlPxEC.

[21] Kolmogoroff A (1931) Uber die analytischen Methoden in der Wahrscheinlichkeitsrechnung. Mathematische Annalen 104: 415–458.

[22] Feller W (1940) On the Integro-Differential Equations of Purely Discontinuous Markoff Processes. Transactions of the American Mathematical Society 48.

[23] Feller W (1949) On the theory of stochastic processes, with particular reference to applications. Proc Berkeley Sympos Math Statist and Probability 1946: 403–432 (1949).

[24] Roxin A, Brunel N, Hansel D, Mongillo G, van Vreeswijk C (2011) On the distribution of firing rates in networks of cortical neurons. J Neurosci 31: 16217–16226. 22072673 10.1523/JNEUROSCI.1677-11.2011 PMC6633220

[25] Stein R (1965) A theoretical analysis of neuronal variability. Biophysical Journal 5: 173–194. 14268952 10.1016/s0006-3495(65)86709-1 PMC1367716

[26] Wilbur W, Rinzel J (1982) An analysis of Stein's model for stochastic neuronal excitation. Biological Cybernetics 45: 107–114. 7138956 10.1007/BF00335237

[27] Knight B (2000) Dynamics of encoding in neuron populations: some general mathematical features. Neural Computation 12: 473–518. 10769319 10.1162/089976600300015673

[28] Sirovich L, Omurtag A, Knight BW (2000) Dynamics of Neuronal Populations: The Equilibrium Solution. SIAM Journal on Applied Mathematics 60: 2009–2028.

[29] Omurtag A, Knight BW, Sirovich L (2000) On the simulation of large populations of neurons. J Comput Neurosci 8: 51–63. 10798499 10.1023/a:1008964915724

[30] Hohn N, Burkitt AN (2001) Shot noise in the leaky integrate-and-fire neuron. Phys Rev E 63: 031902. 10.1103/PhysRevE.63.031902 11308673

[31] Kuhn A, Aertsen A, Rotter S (2003) Higher-order statistics of input ensembles and the response of simple model neurons. Neural Computation 15: 67–101. 12590820 10.1162/089976603321043702

[32] Sirovich L (2003) Dynamics of neuronal populations: eigenfunction theory; some solvable cases. Network 14: 249–272. 12790184

[33] Richardson MJE, Gerstner W (2006) Statistics of subthreshold neuronal voltage fluctuations due to conductance-based synaptic shot noise. Chaos: An Interdisciplinary Journal of Nonlinear Science 16: 026106. 10.1063/1.2203409 16822038

[34] Wolff L, Lindner B (2008) Method to calculate the moments of the membrane voltage in a model neuron driven by multiplicative filtered shot noise. Physical Review E 77: 041913. 10.1103/PhysRevE.77.041913 18517662

[35] Helias M, Deger M, Diesmann M, Rotter S (2009) Equilibrium and response properties of the integrate-and-fire neuron in discrete time. Frontiers in computational neuroscience 3: 29. 10.3389/neuro.10.029.2009 PMC2805428 20130755

[36] Helias M, Deger M, Rotter S, Diesmann M (2010) Instantaneous non-linear processing by pulse-coupled threshold units. PLoS Comput Biol 6: e1000929. 20856583 10.1371/journal.pcbi.1000929 PMC2936519

[37] Helias M, Deger M, Rotter S, Diesmann M (2011) Finite post synaptic potentials cause a fast neuronal response. Front Neurosci 5: 19. 21427776 10.3389/fnins.2011.00019 PMC3047297

[38] Nykamp D, Tranchina D (2000) A population density approach that facilitates large-scale modeling of neural networks: Analysis and an application to orientation tuning. Journal of Computational Neuroscience 8: 19–50. 10798498 10.1023/a:1008912914816

[39] Richardson MJE, Swarbrick R (2010) Firing-Rate Response of a Neuron Receiving Excitatory and Inhibitory Synaptic Shot Noise. Phys Rev Lett 105: 178102. 21231083 10.1103/PhysRevLett.105.178102

[40] Fourcaud-Trocmé N, Hansel D, Van Vreeswijk C, Brunel N (2003) How spike generation mechanisms determine the neuronal response to fluctuating inputs. The Journal of neuroscience 23: 11628–11640. 14684865 10.1523/JNEUROSCI.23-37-11628.2003 PMC6740955

[41] Millman D, Mihalas S, Kirkwood A, Niebur E (2010) Self-organized criticality occurs in nonconservative neuronal networks during Up states. Nat Phys 6: 801–805. 21804861 10.1038/nphys1757 PMC3145974

[42] Markram H, Tsodyks M (1996) Redistribution of synaptic efficacy between neocortical pyramidal neurons. Nature 382: 807–810. 8752273 10.1038/382807a0

[43] Burkitt A (2006) A Review of the Integrate-and-fire Neuron Model: I. Homogeneous Synaptic Input. Biological Cybernetics 95: 1–19. 16622699 10.1007/s00422-006-0068-6

