# Supplementary material for: The Influence of Synaptic Weight Distribution on Neuronal Population Dynamics
Source: PLoS Comput Biol. 2013 Oct 24;9(10):e1003248. doi: 10.1371/journal.pcbi.1003248 (PMC3808453; doi:10.1371/journal.pcbi.1003248)
Supplement: Table S1 — Computational times. Table shows the simulation times in (s) with NEST and DiPDE for different choices of time step dt. The middle four columns correspond to simulation times with different numbers of neurons used in the NEST simulations. The last column shows simulation times for DiPDE with numbers in parantheses representing the number of non-zero elements for a given time step . (PDF) [file pcbi.1003248.s015.pdf]

dt (ms) \ N	$10,000$	$20,000$	$50,000$	$100,000$	DiPDE (nnz)
0.1	8.4	17.1	42.7	82.8	7.5 (42,707)
0.5	2.8	5.6	13.9	28.8	0.5 (5,656)
1.0	2.1	4.3	10.8	21.8	0.2 (1,782)
