# Supplementary material for: The Influence of Synaptic Weight Distribution on Neuronal Population Dynamics
Source: PLoS Comput Biol. 2013 Oct 24;9(10):e1003248. doi: 10.1371/journal.pcbi.1003248 (PMC3808453; doi:10.1371/journal.pcbi.1003248)
Supplement: Table S2 — Differentiating synaptic weight distributions matched for mean input current. Table shows the number of independent recordings of sub-threshold steady state membrane potential required to differentiate between synaptic distributions matched for mean input current. The values above the diagonal are the sample sizes needed for p = 0.01, and below the diagonal are the sizes for p = 0.05. (PDF) [file pcbi.1003248.s016.pdf]

Distributions	Delta	Gaussian	Exponential	Lognormal	Bimodal	Power law
Delta	Inf	1,853	553	395	23	37
Gaussian	1,291	Inf	2,632	1,350	27	44
Exponential	385	1,833	Inf	16,550	31	53
Lognormal	276	940	11,523	Inf	32	55
Bimodal	16	19	22	23	Inf	535
Power law	26	31	37	39	373	Inf
