# Supplementary material for: The Influence of Synaptic Weight Distribution on Neuronal Population Dynamics
Source: PLoS Comput Biol. 2013 Oct 24;9(10):e1003248. doi: 10.1371/journal.pcbi.1003248 (PMC3808453; doi:10.1371/journal.pcbi.1003248)
Supplement: Table S3 — Differentiating synaptic weight distributions matched for drift and diffusion. Table shows the number of independent recordings of sub-threshold steady state membrane potential required to differentiate between synaptic distributions matched for drift and diffusion. The values above the diagonal are the sample sizes needed for p = 0.01, and below the diagonal are the sizes for p = 0.05. (PDF) [file pcbi.1003248.s017.pdf]

Distributions	Delta	Gaussian	Exponential	Lognormal	Bimodal	Power law
Delta	Inf	48,850	31,326	6,730	85	44
Gaussian	34,012	Inf	60,638	7,715	80	42
Exponential	21,749	42,219	Inf	17,409	83	43
Lognormal	4,686	5,372	12,121	Inf	93	46
Bimodal	59	56	58	65	Inf	526
Power law	31	29	30	32	367	Inf
