# Supplementary material for: The Influence of Synaptic Weight Distribution on Neuronal Population Dynamics
Source: PLoS Comput Biol. 2013 Oct 24;9(10):e1003248. doi: 10.1371/journal.pcbi.1003248 (PMC3808453; doi:10.1371/journal.pcbi.1003248)
Supplement: Table S4 — Dynamical ranges. Table shows the upper (90%) and lower (10%) limits of steady state output firing rates and dynamical ranges (ratio of upper and lower steady state output firing rates) for synaptic weight distributions (as in Figure (2)) matched for mean input synaptic current. Dynamical range increases as the distributions get heavier-tailed. (PDF) [file pcbi.1003248.s018.pdf]

Distribution	Lower f_l (Hz)	Upper f_u (Hz)	Dyn. Range (f_u/f_l)
delta	1,639.4	16,761	10.22
Gaussian	1,393.9	16,602	11.91
exponential	1,194.2	16,000	13.40
lognormal	1,054.5	16,000	15.17
bimodal	432.6	13,768	31.83
power law	405.0	12,823	31.66
