# Supplementary material for: The Influence of Synaptic Weight Distribution on Neuronal Population Dynamics
Source: PLoS Comput Biol. 2013 Oct 24;9(10):e1003248. doi: 10.1371/journal.pcbi.1003248 (PMC3808453; doi:10.1371/journal.pcbi.1003248)
Supplement: Table S5 — Relative excitabilities. Table shows the maximum relative excitability for synaptic weight distributions matched for mean input current, without and with synaptic depression. Heavier-tailed distributions lead to smaller changes in relative excitability. (PDF) [file pcbi.1003248.s019.pdf]

Distribution	Max rel. exc (without syn dep)	Max rel. exc (with syn dep)
delta	2.95	1.97
Gaussian	3.00	1.68
exponential	2.92	1.52
lognormal	2.88	1.45
bimodal	1.32	1.07
power law	1.76	1.08
