# Supplementary material for: The Influence of Synaptic Weight Distribution on Neuronal Population Dynamics
Source: PLoS Comput Biol. 2013 Oct 24;9(10):e1003248. doi: 10.1371/journal.pcbi.1003248 (PMC3808453; doi:10.1371/journal.pcbi.1003248)
Supplement: Text S1 — A brief review of stochastic processes focusing on elements needed for this study. (PDF) [file pcbi.1003248.s020.pdf]

Text S1

S1 Stochastic processes

To help readers, a short review of stochastic processes focusing on elements needed for this study is presented following *'Stochastic Processes in Physics and Chemistry'* by N.G. van Kampen [40]. The study of stochastic dynamical systems is based on the Chapman-Kolmogorov equation [41]. For a continuous state-space for the variable y and time ordering $t_3 \geq t_2 \geq t_1$, this equation reads

$$P(y_3, t_3|y_1, t_1) = \int dy_2 P(y_3, t_3|y_2, t_2)P(y_2, t_2|y_1, t_1) \tag{33}$$

Here $P(y_j, t_j|y_i, t_i)$ represents the conditional probability that a system makes a transition to the state y_j at time t_j if it was in state y_i at time t_i. The Chapman-Kolmogorov equation embodies the Markov assumption in that only the present state is necessary to compute the future state; the past is irrelevant to this computation. When the state space is discrete, equation (33) can be equivalently written as,

$$P(i, s|k, t) = \sum_j P(i, s|j, \delta)P(j, \delta|k, t) \tag{34}$$

Starting with the theory of discrete time Markov chains governed by equation (34), Kolmogorov derived so-called forward and backward differential equations for two kinds of continuous time Markov processes [41–43], depending on the assumed behavior over small intervals of time :
i) 'Jump processes' for which in a small time interval there is an overwhelming probability that the state will remain unchanged; however if it changes, the change maybe radical.
ii) Processes such as those which are represented by diffusion and Brownian motion, for which some change will occur in any interval of time, however small; it is certain, however, that changes during small time intervals will also be small.

For a one-dimensional system, the generic forward differential equation can be written as,

$$\begin{aligned} \frac{\partial}{\partial t}P(z, t|y, s) &= -\frac{\partial}{\partial z}\left[A(z, t)P(z, t|y, s)\right] + \frac{1}{2}\frac{\partial^2}{\partial z^2}\left[B(z, t)P(z, t|y, s)\right] \\ &\quad + \int dx \left[W(z|x, t)P(x, t|y, s) - W(x|z, t)P(z, t|y, s)\right] \end{aligned} \tag{35}$$

The conditional quantity $W(x|z, t)$ represents the transition probability for discrete jumps in state space. In this equation, the following definitions are assumed with $\epsilon = |x - z|$,

$$\begin{aligned} \lim_{\Delta t \to 0} \frac{P(x, t + \Delta t|z, t)}{\Delta t} &= W(x|z, t) \\ \lim_{\Delta t \to 0} \frac{1}{\Delta t}\int_z^{z+\epsilon} dx(x - z)P(x, t + \Delta t|z, t) &= A(z, t) + O(\epsilon) \\ \lim_{\Delta t \to 0} \frac{1}{\Delta t}\int_z^{z+\epsilon} dx(x - z)^2 P(x, t + \Delta t|z, t) &= B(z, t) + O(\epsilon) \end{aligned} \tag{36}$$

The appropriate adjoint of equation (35) would then correspond to the backward differential equation.

For $A(z,t) = 0$ and $B(z,t) = 0$, equation (35) is known as a 'master equation' [40]. The system admits solutions which are constants separated by finite jumps at discrete time points with density $W(z|x,t)$ and correspond to 'jump processes'. On the other hand if $W(z|x,t) = 0$, then equation (35) reduces to the Fokker-Planck equation. The process has continuous paths and the quantities A and B correspond to the drift and diffusion respectively.

A deterministic system subject to external Gaussian white noise is described by a stochastic differential equation (SDE) known as the Langevin equation:

$$\frac{dy}{dt} = A(y) + B(y)\xi(t) \tag{37}$$

where $\xi(t)$ represents Gaussian white noise, with average $<\xi(t)>= 0$ and $<\xi(t)\xi(t')>= \delta(t-t')$.

When $B(y) = 1$, the noise is 'additive' and it can be shown that equation (37) is equivalent to the Fokker-Planck equation,

$$\frac{\partial}{\partial t}P(y,t) = -\frac{\partial}{\partial y}\left[A(y)P(y,t)\right] + \frac{1}{2}\frac{\partial^2}{\partial y^2}P(y,t) \tag{38}$$

When applied to neuronal systems, if all the currents which depend on membrane potential are included in the threshold nonlinearity, the noise is additive. However in the case of conductance based synapses, synaptic currents depend on the membrane potential, so that multiplicative noise is present ($B(y) \neq 1$) and two distinct formulations (Ito and Stratonovich calculus) exist for the description of the SDE.
