# Supplementary material for: The Influence of Synaptic Weight Distribution on Neuronal Population Dynamics
Source: PLoS Comput Biol. 2013 Oct 24;9(10):e1003248. doi: 10.1371/journal.pcbi.1003248 (PMC3808453; doi:10.1371/journal.pcbi.1003248)
Supplement: Text S2 — Comparison of DiPDE and Fokker-Planck formalisms. (PDF) [file pcbi.1003248.s021.pdf]

Text S2

S2 Comparison of DiPDE and Fokker-Planck

As described above, the Fokker-Planck equation naturally arises in the description of continuous stochastic processes. In the context of neuronal systems, the Fokker-Planck equation describes the time evolution of the probability $p(v, t)$ that a neuron has membrane potential v at time t. The synaptic activity is abstracted to contribute to the drift and diffusion terms in the equation. Thus, the entire distribution of synaptic weights can be completely characterized by only *two* quantities. The Fokker-Planck formalism is based on a Wiener process, which is a continuous stochastic process. In an infinitesimal time step, the random process will keep the random variable v in an infinitesimal interval from its starting value. In contrast, with DiPDE we maintain the size of the synaptic weight to be finite. However its probability of activation is considered infinitesimal for each infinitesimal time step.

This leads to marked differences between Fokker-Planck and DiPDE for small numbers of relatively large synaptic inputs and helps in the exploration of generic distributions of synaptic weights. However, in the limit of a large number of relatively small synaptic inputs, it can be expected that solutions of equation (10) converge to those obtained from the Fokker-Planck equation. Figure (S1) shows a comparison of results obtained from each method for many small equal synapses. It is evident that all three methods converge to the same result as expected.

The DiPDE numerical solution provides an equilibrium firing rate of $f_o = 17.9$ Hz and a transient firing time $t_s = 17.0$ ms, while Fokker-Planck results in an equilibrium firing rate of $f_o = 17.4$ Hz and a transient firing time of $t_s = 18.1$ ms.
