# Supplementary material for: The Influence of Synaptic Weight Distribution on Neuronal Population Dynamics
Source: PLoS Comput Biol. 2013 Oct 24;9(10):e1003248. doi: 10.1371/journal.pcbi.1003248 (PMC3808453; doi:10.1371/journal.pcbi.1003248)
Supplement: Text S3 — Computational complexity with DiPDE. (PDF) [file pcbi.1003248.s022.pdf]

Text S3

S3 Computational complexity with DiPDE

For a given time step dt and number N of LIF neurons to be simulated, the computational cost involved with a NEST simulation for a feed-forward network is $O(N/dt)$. The $1/dt$ dependence follows from the fact that the number of time steps involved in the simulation $N_t \sim 1/dt$. In our DiPDE simulations, there is no reference to a fixed number of neurons in the population; we solve for the probability distribution $p(v,t)$ for the neuronal membrane potential to be at some v between 0 and θ at time t. The computational cost for a DiPDE simulation with time step dt is dominated by the synaptic input step. As outlined in (Methods: Numerical Solutions), this step involves multiplying a $(N_v \times 2N_v)$ transition matrix T with a $(N_v \times 1)$ vector of the probability distribution $p(v, t_k)$ for the membrane potential at time t_k. As in equation (15), $N_v = -\frac{\tau_m}{dt} \ln\left(1 - e^{-dt/\tau_m}\right)$ is the number of voltage bin-edges generated with our geometric binning scheme. Thus, the computational cost for a DiPDE simulation of a feed-forward network with external input rate f determined by a homogeneous Poisson process scales asymptotically as $O(N_v^2/dt)$. For a δ-function or bimodal distribution of synaptic weights, the transition matrix T is sparse. In such cases, the computational cost scales asymptotically as $O(nnz/dt)$ where $nnz \sim N_v$ is the number of non-zero elements in the sparse transition matrix T.
Table (S1) shows the total simulation times using NEST (with different numbers of neurons N) and DiPDE for different choices of time step dt, for the feed-forward network of Fig. (1). Note that the transition matrix T in this case is a $N_v \times 2N_v$ sparse matrix which keeps track of the effect of super-threshold synaptic inputs. Ignoring the effect of the excess synaptic input and re-setting the membrane potential to zero would lead to a $N_v \times N_v$ sparse matrix.
With an unconditionally stable numerical scheme to solve the Fokker-Planck equation, the computational cost involved would scale asymptotically as $O(N_v^{'}/dt')$, where $N_v^{'}$ is the number of uniform bins used to discretize the voltage between 0 and θ and dt' is the time step used.
