# Supplementary material for: The Influence of Synaptic Weight Distribution on Neuronal Population Dynamics
Source: PLoS Comput Biol. 2013 Oct 24;9(10):e1003248. doi: 10.1371/journal.pcbi.1003248 (PMC3808453; doi:10.1371/journal.pcbi.1003248)
Supplement: Text S4 — p-values for differences between sub-threshold steady-state membrane potential distributions obtained from different synaptic weight distributions. (PDF) [file pcbi.1003248.s023.pdf]

Text S4

S4 Differences between sub-threshold steady-state membrane potential distributions

Using a Kolmogorov-Smirnov test for the sub-threshold steady-state membrane potential distributions (null hypothesis is that distributions are the same), p-values can be obtained for the differences between these distributions.

1. (Fig.1e) Current-based synapses with $w = 4.867$ mV and $f = 100$ Hz.
   a) FP vs. simulations: p=7.38e-85
   b) DiPDE vs. simulations: p=0.524
   c) FP vs. DiPDE: A sample size of $N = 421$ neurons in the population for $p = 0.05$ and $N = 605$ for $p = 0.01$.

2. (Fig.S1e) Current-based synapses with $w = 0.974$ mV and $f = 1,000$ Hz.
   a) FP vs. simulations: p=0.0874
   b) DiPDE vs. simulations: p=0.9934
   c) FP vs. DiPDE: A sample size of $N = 27,227$ neurons for $p = 0.05$ and $N = 39,104$ for $p = 0.01$.

3. (Fig.S3d) Conductance-based synapses with $w = 4.749$ mV and $f = 100$ Hz.
   a) DiPDE vs. simulations: p=0.3967

4. For steady state membrane potential distributions obtained from synaptic distributions matched for mean input current shown in Fig. (2e), there is no empirical sampled data, so the comparison here is the minimum number of independent recordings of sub-threshold steady state membrane potential recordings required to distinguish between the two distributions (Supplement Table (S2)). Larger numbers mean the distributions are more similar. The values above the diagonal are the sample sizes needed for p=0.01, and below the diagonal are the sizes for p=0.05.

5. The same arguments as above can be applied to steady state membrane potential distributions obtained from synaptic distributions matched for drift and diffusion shown in Fig. (S7b). The minimum number of independent recordings required to differentiate two synaptic weight distributions are listed in Supplement Table (S3). Larger numbers mean the distributions are more similar. The values above the diagonal are the sample sizes needed for p=0.01, and below the diagonal are the sizes for p=0.05.
